# Prostaglandin E_2_ promotes intestinal inflammation via inhibiting microbiota-dependent regulatory T cells

**DOI:** 10.1126/sciadv.abd7954

**Published:** 2021-02-12

**Authors:** Siobhan Crittenden, Marie Goepp, Jolinda Pollock, Calum T. Robb, Danielle J. Smyth, You Zhou, Robert Andrews, Victoria Tyrrell, Konstantinos Gkikas, Alexander Adima, Richard A. O’Connor, Luke Davies, Xue-Feng Li, Hatti X. Yao, Gwo-Tzer Ho, Xiaozhong Zheng, Amil Mair, Sonja Vermeren, Bin-Zhi Qian, Damian J. Mole, Konstantinos Gerasimidis, Jürgen K. J. Schwarze, Richard M. Breyer, Mark J. Arends, Valerie B. O’Donnell, John P. Iredale, Stephen M. Anderton, Shuh Narumiya, Rick M. Maizels, Adriano G. Rossi, Sarah E. Howie, Chengcan Yao

**Affiliations:** 1 Centre for Inflammation Research, Queen’s Medical Research Institute, The University of Edinburgh, Edinburgh EH16 4TJ, UK.; 2 SRUC Veterinary Services, Scotland’s Rural College, Easter Bush Estate EH26 0PZ, UK.; 3 Wellcome Centre for Molecular Parasitology, Institute for Infection, Immunity and Inflammation, University of Glasgow, Glasgow G12 8TA, UK.; 4 Systems Immunity University Research Institute and Division of Infection and Immunity, Cardiff University, Cardiff CF14 4XN, UK.; 5 Human Nutrition, School of Medicine, Dentistry and Nursing, University of Glasgow, Glasgow G31 2ER, UK.; 6 MRC Centre for Reproductive Health, Queen’s Medical Research Institute, The University of Edinburgh, Edinburgh EH16 4TJ, UK.; 7 Department of Veterans Affairs, Tennessee Valley Health Authority, and Department of Medicine, Vanderbilt University Medical Center, Nashville, TN, USA.; 8 Division of Pathology, Cancer Research UK Edinburgh Centre, The University of Edinburgh, Institute of Genetics and Molecular Medicine, Edinburgh EH4 2XR, UK.; 9 Senate House, University of Bristol, Bristol BS8 1TH, UK.; 10 Alliance Laboratory for Advanced Medical Research and Department of Drug Discovery Medicine, Medical Innovation Center, Kyoto University Graduate School of Medicine, Kyoto 606-8507, Japan.

## Abstract

The gut microbiota fundamentally regulates intestinal homeostasis and disease partially through mechanisms that involve modulation of regulatory T cells (T_regs_), yet how the microbiota-T_reg_ cross-talk is physiologically controlled is incompletely defined. Here, we report that prostaglandin E_2_ (PGE_2_), a well-known mediator of inflammation, inhibits mucosal T_regs_ in a manner depending on the gut microbiota. PGE_2_ through its receptor EP4 diminishes T_reg_-favorable commensal microbiota. Transfer of the gut microbiota that was modified by PGE_2_-EP4 signaling modulates mucosal T_reg_ responses and exacerbates intestinal inflammation. Mechanistically, PGE_2_-modified microbiota regulates intestinal mononuclear phagocytes and type I interferon signaling. Depletion of mononuclear phagocytes or deficiency of type I interferon receptor diminishes PGE_2_-dependent T_reg_ inhibition. Together, our findings provide emergent evidence that PGE_2_-mediated disruption of microbiota-T_reg_ communication fosters intestinal inflammation.

## Teaser

PGE_2_ inhibits T_regs_ and promotes intestinal inflammation through actions on mononuclear phagocytes and the gut microbiota.

## INTRODUCTION

Inflammatory bowel disease (IBD) is a chronic inflammatory disorder of the intestine that causes abdominal pain, diarrhea, bleeding, and increased risk of intestinal cancer. There are two main subtypes of IBD, i.e., Crohn’s disease (CD) and ulcerative colitis. Multiple factors including lifestyle (e.g., smoking, diet, medication, and psychological state), environmental risk factors (e.g., infections and air pollution), genetic and epigenetic alterations, and host immune functions can potentially trigger the development and progression of IBD (*1*–*2*). The gut microbiota plays a critical role in maintaining health of the host. Dysfunction of this symbiosis may result in development of various human diseases such as IBD, metabolic syndrome, infections, allergy, and cancer (*3*–*4*). Interplay between the host and gut microbiota controls intestinal homeostasis and inflammatory responses through mechanisms that involve modulation of gut-resident regulatory T cells (T_regs_), which express the transcription factor, forkhead box P3 (Foxp3) (*5*–*8*). Dysregulation of intestinal T_regs_ is implicated in the pathogenesis of IBD (*9*). Microbial antigens, metabolites [e.g., vitamins and short-chain fatty acids (SCFAs)], and signaling molecules released during tissue damage (e.g., alarmins) contribute to the induction of distinct intestinal T_reg_ subsets, which play critical roles in intestinal homeostasis and control mucosal inflammation (*10*–*13*). However, the mechanisms that negatively regulate microbiota-T_reg_ cross-talk for IBD pathogenesis are incompletely studied.

Prostaglandins (PGs) are bioactive lipid mediators that are generated from arachidonic acid via cyclooxygenases (COXs) and specific PG synthases (*14*). The PG family has five members including PGE_2_, PGD_2_, PGF_2α_, PGI_2_, and thromboxane A_2_ (TXA_2_). PGs signal in an autocrine and/or paracrine manner through their distinct G protein–coupled receptors including receptors EP1 to EP4, PGD_2_ receptors DP1 and DP2, PGF_2α_ receptor FP, PGI_2_ receptor IP, and TXA_2_ receptor TP. PGE_2_ is present in most tissues at biologically functional nanomolar levels in the steady state, and its levels are increased at the sites of inflammation (*14*–*16*). Nonsteroidal anti-inflammatory drugs (NSAIDs), such as aspirin and indomethacin, are widely used to reduce pain, fever, and inflammation by inhibiting COX activities and therefore decreasing PG production. However, NSAIDs are generally avoided for individuals who have gut conditions due to the gastrointestinal adverse effects (*17*). This is because PGE_2_ plays critical roles in maintaining the gut epithelium, protecting against acute damage, and facilitating regeneration after injury through actions on various cell types including macrophages, epithelial, stromal, and innate lymphoid cells (*18*–*21*).

Genome-wide association studies have revealed that polymorphisms in the *PTGER4* gene (encoding human PGE_2_ receptor EP4) are associated with overexpression of EP4 and a more severe disease phenotype in patients with IBD (*22*–*24*). Moreover, variants in the *PTGER4* gene exert a significant association with CD, in third place among all susceptible genetic loci after variants in *NOD2* and *IL23R* genes (*25*). These findings raised a possibility that PGE_2_-EP4 signaling may also participate in the pathogenesis of intestinal inflammation despite its protective actions on the epithelium. We and others have recently found that PGE_2_ plays key roles in immune-related chronic inflammatory diseases in rodents and humans, for example, multiple sclerosis, rheumatoid arthritis, and inflammatory skin disorders, through promoting interferon-γ (IFN-γ)–producing T helper 1 (T_H_1) cells and interleukin-17 (IL-17)–producing T_H_17 cells by induction of key cytokine receptors IL-12Rβ2 and IL-23R, respectively (*26*–*29*). However, the action of PGE_2_ on T_reg_ responses, especially in the intestine, remains unknown. In this study, we investigated the roles for endogenous PGE_2_ in regulation of mucosal T_reg_ responses and intestinal inflammation. We demonstrate that PGE_2_ down-regulates intestinal T_reg_ responses by affecting the composition of the gut microbiota and modulating the function of mononuclear phagocytes (MNPs).

## RESULTS

### Production of PGs and expression of their receptors in the intestine

**Fig. 1:**
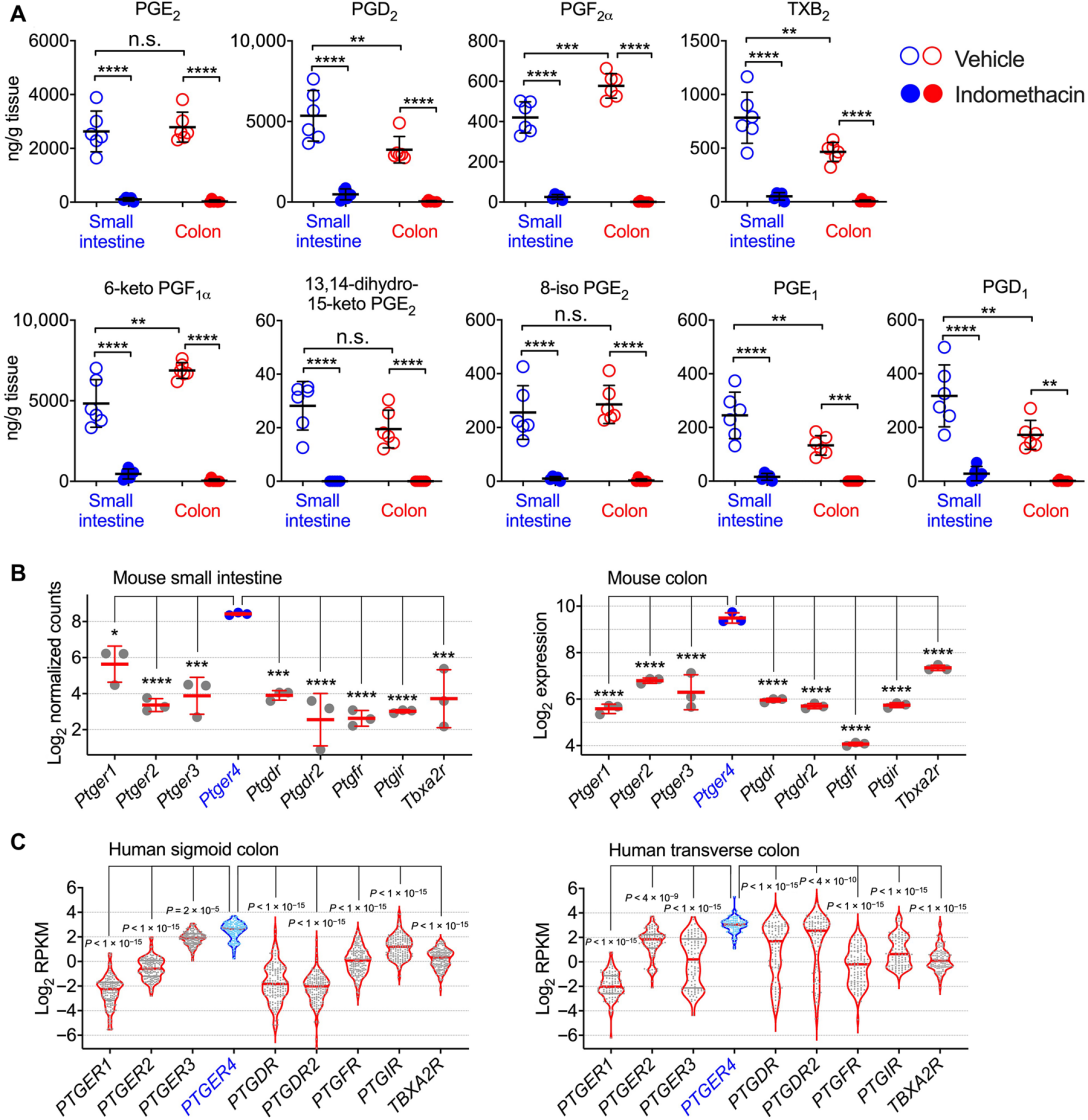
Expression of PGs and their receptors in the intestine. (**A**) Levels of PGs and their metabolites in small intestines and colons from mice administered with indomethacin or vehicle control in drinking water for 5 days (*n* = 6 each group) as measured by lipidomic analysis. (**B**) Gene expression of PG receptors in mouse small and large intestines. Microarray gene expression data of normal C57BL/6 mouse colons (*n* = 3) were retrieved from the Gene Expression Omnibus (GEO) dataset GSE31106. RNA sequencing (RNA-seq) data of normal C57BL/6 mouse small intestines (*n* = 3) were retrieved from the GEO dataset GSE97371. (**C**) Gene expression of PG receptors in human sigmoid (*n* = 149) and transverse (*n* = 104) colon biopsy samples of healthy individuals. RNA-seq data were downloaded from the Genotype-Tissue Expression project database and analyzed using Python 3.7.0. RPKM, reads per kilobase of transcript. Each scatter dot plot in bar graphs represents data from one mouse (A and B) or individual (C). Data shown as means ± SD (A and B) or presented as violin bars with scatter plots (C) are analyzed by analysis of variance (ANOVA) with post hoc Holm-Sidak’s multiple comparisons test. *P < 0.05, **P < 0.01, ***P < 0.001, and ****P < 0.0001. n.s., not significant.

First, we examined whether the intestine tissues physiologically produce PGs and whether inhibition of COXs reduces PG levels in the steady state. We administered naïve wild-type (WT) C56BL/6 mice with a nonselective COX inhibitor, indomethacin, at the dose of 5 mg/kg per day (equaling to ~30 mg/day for an adult human weighing 75 kg) or vehicle control [0.5% ethanol (EtOH)] in drinking water. Intestine tissues were collected on day 5 for measuring levels of PGs by lipidomic analysis. We observed that healthy small and large intestines in control mice produced high levels (i.e., hundreds to thousands nanogram per gram dry tissue) of PGs including PGE_2_, PGD_2_, PGF_2α_, TXA_2_ metabolite (i.e., TXB_2_), and PGI_2_ metabolite (i.e., 6-keto PGF_1α_) (Fig. 1A and fig. S1). Small intestine produced more PGD_2_ and TXB_2_, but less PGF_2α_ and 6-keto PGF_1α_, than the colon, while both intestinal tissues had comparable levels of PGE_2_ and its metabolite (13,14-dihydro-15-keto PGE_2_) or epimer (8-iso PGE_2_) (Fig. 1A). Administration of indomethacin nearly completely inhibited production of all PGs (e.g., PGE_2_, PGD_2_, and PGF_2α_) and their inactive metabolites, i.e., 15-keto PGE_2_, 15-keto PGD_2_, 15-keto PGF_2α_, 6-keto-PGF_1α_, and TXB_2_ in both small and large intestines (Fig. 1A). For example, indomethacin reduced PGE_2_ levels by ~96% (from 2628 to 105 ng/g of dry tissue) in small intestines and by ~99% (from 2789 to 29 ng/g of dry tissue) in colons (Fig. 1A). Reanalysis of public datasets (*30*) revealed that both mouse and human intestines express EP4 genes (i.e., *Ptger4* and *PTGER4*, respectively) at the levels remarkedly higher than other PG receptors (Fig. 1, B and C), indicating that the PGE_2_-EP4 pathway may play a more important role than other PG signaling in the intestine.

### Increase of intestinal T_regs_ by COX inhibition

**Fig. 2:**
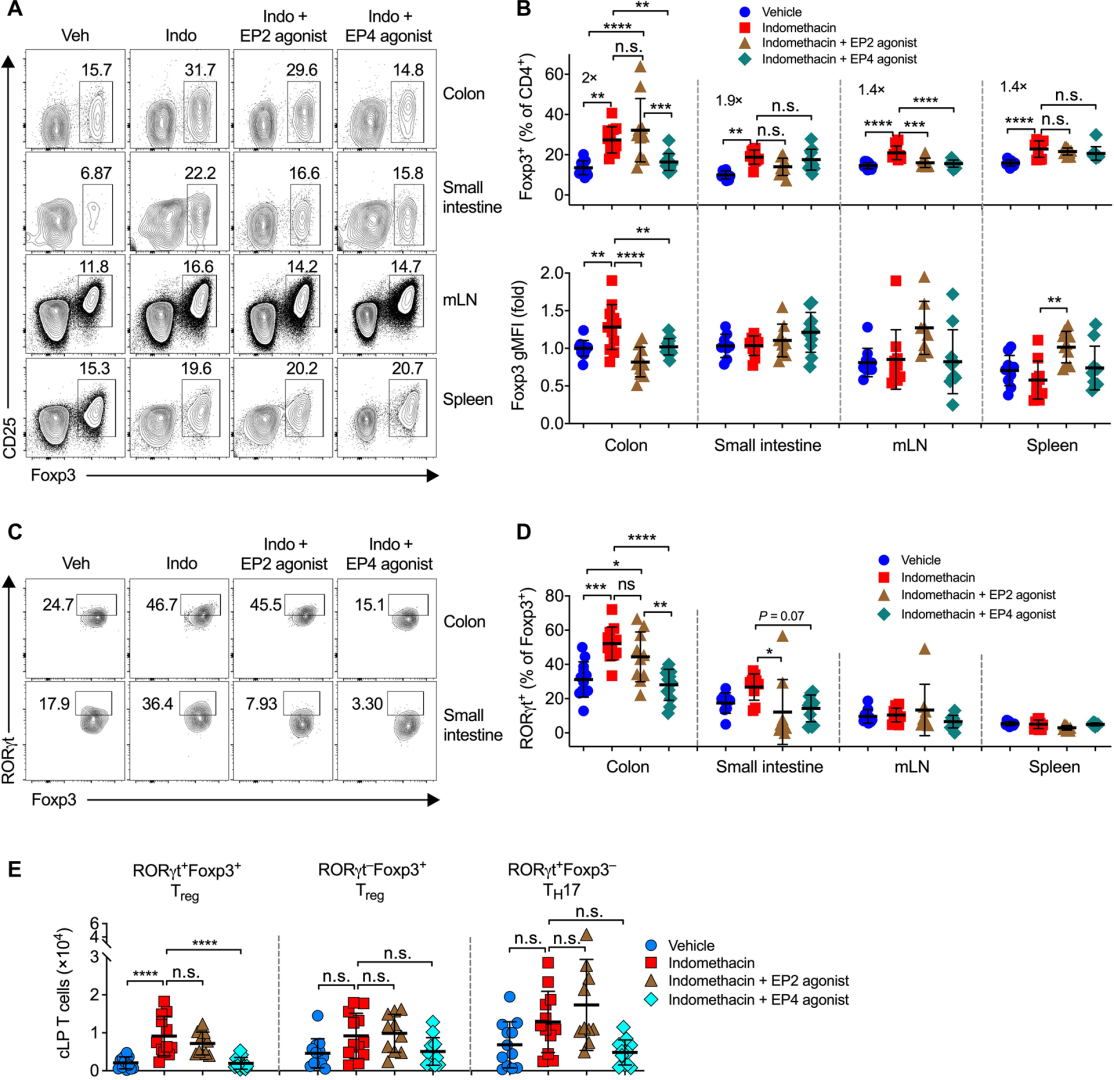
PGE_2_-EP4 signaling inhibits intestinal T_regs_ in the steady state. (**A**) Representative flow cytometry plots of Foxp3^+^ T_regs_ gated on live CD45^+^CD3^+^CD4^+^ T cells in colon and small intestinal LP, mLN, and spleen from C57BL/6 mice that were treated with vehicle (Veh) or indomethacin (Indo) in drinking water and injected intraperitoneally with an EP2 agonist, Butaprost, or an EP4 agonist, L-902,688 (*n* = 8 to 12). (**B**) Percentages of Foxp3^+^ T_regs_ (top) and geometric MFI (gMFI) of Foxp3 (bottom). (**C**) Representative flow cytometry dot plots of RORγt expression among Foxp3^+^ T_regs_ in colon and small intestinal LP. (**D**) Percentages of RORγt expression by Foxp3^+^ T_regs_. (**E**) Absolute numbers of RORγt^+^Foxp3^+^ T_regs_, RORγt^−^Foxp3^+^ T_regs_, RORγt^+^Foxp3^−^ T_H_17 cells in colon LP (cLP). Each scatter dot plot in bar graphs represents data from one mouse. Data shown as means ± SD are pooled from four independent experiments and analyzed by ANOVA with post hoc Holm-Sidak’s multiple comparisons test (B, D, and E). *P < 0.05, **P < 0.01, ***P < 0.001, and ****P < 0.0001. n.s., not significant.

To test whether endogenous PGs regulate intestinal T_regs_ in the steady state, we administered WT mice with indomethacin in drinking water and analyzed T_regs_ in various organs including colons, small intestines, mesenteric lymph nodes (mLNs), and spleens. Indomethacin treatment increased the accumulation of Foxp3^+^ T_regs_ in all of these tissues with greater effects in the intestinal lamina propria (LP) (by ~2-fold in the colon and small intestine) than that in the mLN and spleen (both by ~1.4-fold) (Fig. 2, A and B). Furthermore, indomethacin significantly increased mean fluorescence intensity (MFI) of Foxp3 among Foxp3^+^ T_regs_ in the colon (Fig. 2B), suggesting that inhibition of endogenous PG biosynthesis not only increases intestinal T_reg_ frequencies but also enhances Foxp3 expression at the single-cell level. We also administered mice with indomethacin at lower doses of 1 to 2 mg/kg of body weight per day, which is the equivalent of ~6 to 12 mg/day for adults weighing 75 kg, levels known not to induce intestinal damage in human. Similarly, low doses of indomethacin still increased intestinal Foxp3^+^ T_regs_ (fig. S2).

It has been recently reported that a subpopulation of intestinal T_regs_ that express the transcription factor retinoid-related orphan receptor gamma t (RORγt), namely, RORγt^+^Foxp3^+^ T_regs_, inhibit intestinal inflammation with greater suppressive potential than RORγt^−^Foxp3^+^ T_regs_ (*6*, *7*, *31*). We therefore examined the effects of endogenous PGs on RORγt^+^Foxp3^+^ T_regs_ and found that administration of indomethacin markedly increased the percentages of the RORγt^+^Foxp3^+^ subpopulation among Foxp3^+^ T_regs_ in the colon, but not in the mLN or spleen (Fig. 2, C and D). Furthermore, indomethacin also boosted absolute numbers of colonic RORγt^+^Foxp3^+^ T_regs_, but not the numbers of RORγt^−^Foxp3^+^ T_regs_ or RORγt^+^Foxp3^−^ T_H_17 cells (Fig. 2E). These results indicate that inhibition of endogenous PGs increases the accumulation of intestinal T_regs_.

### Reversion of COX inhibition–dependent increase in intestinal T_regs_ by EP4 agonism

As indomethacin inhibits all PG production, we next examined whether PGE_2_ and its receptors control intestinal T_regs_. We coadministered mice with indomethacin together with a selective EP2 agonist (Butaprost) or selective EP4 agonists (L-902,688). We found that increase in colonic T_regs_ by indomethacin was prevented by the EP4 agonist in colons and mLNs, while the EP2 agonist only reduced T_regs_ in mLNs (Fig. 2, A and B), indicating that PGE_2_ suppresses colonic T_reg_ accumulation mainly through EP4. Similarly, coadministration of EP4 agonist notably down-regulated RORγt^+^Foxp3^+^ T_regs_ in the colon and likely in the small intestine, but not in the mLN or spleen (Fig. 2, C and D). Furthermore, selective activation of EP4 also decreased absolute numbers of colonic RORγt^+^Foxp3^+^ T_regs_, but not RORγt^−^Foxp3^+^ T_regs_ or RORγt^+^Foxp3^−^ T_H_17 cells (Fig. 2E). These results indicate that activation of PGE_2_-EP4 signaling overturns NSAID-dependent augmentation of T_regs_ with the greatest potency in the intestine.

To further examine whether exogenous activation of EP4 suppresses colonic T_regs_, we injected the EP4 agonist alone into naïve WT C57BL/6 mice without coadministration of indomethacin. Unlike activation of EP4 in indomethacin-treated mice where all endogenous PG production was inhibited (Fig. 2), administration of EP4 agonist into naïve WT mice where all PG signaling pathways were intact had few effects on colonic T_reg_ accumulation, albeit a trend for reducing the proportion of RORγt^+^Helio^−^ T_regs_ (fig. S3). This may be due to the already high levels of PGE_2_ and other EP4 ligands (e.g., PGE_1_) in naïve intestines (Fig. 1A). There is another possibility that blockade of endogenous PGs by indomethacin disrupted the intestinal epithelial line (*19*), which leads to attachment and translocation of more invasive commensal microbes and in turn increases the accumulation of colonic T_regs_. Coadministration of EP4 agonist prevented indomethacin-dependent epithelial damage and attachment of invasive microbes, resulting in reduction of colonic T_reg_ accumulation. In the naïve intestine without disruption of the epithelial integrity (i.e., without COX inhibition), however, EP4 agonism has thus no influence on T_reg_ accumulation in the colon. Nevertheless, these results collectively indicate that PGE_2_-EP4 signaling has a potency to inhibit intestinal T_reg_ accumulation.

### Involvement of the gut microbiota in PGE_2_ suppression of intestinal T_regs_

**Fig. 3:**
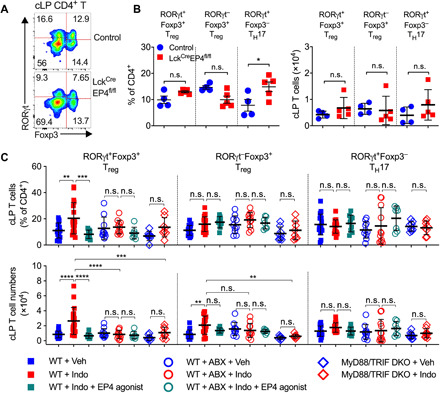
The gut microbiota is involved in PGE_2_-EP4 signaling regulation of intestinal T_regs_. (**A** and **B**) Representative flow cytometry plots (A) and percentages and numbers (B) of RORγt^+^Foxp3^+^ T_regs_, RORγt^−^Foxp3^+^ T_regs_, RORγt^+^Foxp3^−^ T_H_17 cells in colon LP of Lck^Cre^EP4^fl/fl^ (*n* = 5) and control (*n* = 4) mice determined by flow cytometry. (**C**) Percentages and numbers of RORγt^+^Foxp3^+^ T_regs_, RORγt^−^Foxp3^+^ T_regs_, and RORγt^+^Foxp3^−^ T_H_17 cells in colon LP of WT and MyD88/TRIF double knockout (DKO) mice that were treated with vehicle, indomethacin, or indomethacin plus EP4 agonist, L-902,688 (*n* = 7 to 18). Antibiotics (ABX) were administered to WT mice in drinking water for 2 weeks started 1 week before receiving indomethacin or vehicle. Each scatter dot plot in bar graphs represents data from one mouse. Data shown as means ± SD are pooled from two (B) or five (C) experiments and analyzed by ANOVA with post hoc Holm-Sidak’s multiple comparisons test. *P < 0.05, **P < 0.01, ***P < 0.001, and ****P < 0.0001. n.s., not significant.

To examine whether PGE_2_-EP4 signaling inhibits intestinal T_regs_ in the steady state via direct or indirect actions on T cells, we generated Lck-Cre–driven EP4 conditional knockout mice by crossing EP4-flox mice (*32*) to Lck-Cre mice (i.e., Lck^Cre^EP4^fl/fl^ mice) to delete EP4 expression in Lck-expressing T cells. Lck^Cre^EP4^fl/fl^ and control mice had comparable colonic T_reg_ accumulation in the steady state (Fig. 3, A and B), suggesting that PGE_2_ inhibits intestinal T_reg_ accumulation in the steady state independent of EP4 signaling in T cells.

The gut microbiota is crucial for the development of intestinal T_regs_, especially the RORγt^+^Foxp3^+^ T_reg_ subset (*6*, *7*, *31*). We therefore investigated whether the gut microbiota is involved in PGE_2_-dependent control of intestinal T_regs_. We analyzed colonic T_regs_ from WT mice in which the gut microbiota had been depleted by antibiotics, or from MyD88/TRIF double knockout mice deficient in both myeloid differentiation primary response 88 (MyD88) and TRI domain containing adaptor-inducing interferon-beta (TRIF) that were unable to sense microbial signals. Inhibition of endogenous PGE_2_ by indomethacin increased both the frequencies and numbers of colonic T_regs_ (especially the subpopulation of RORγt^+^Foxp3^+^ T_regs_) but not RORγt^+^Foxp3^−^ T_H_17 cells in WT mice that have been treated with vehicle control (Fig. 3C). In contrast, there was no increased accumulation of colonic T_regs_ by indomethacin in antibiotic-treated WT mice nor in those mice with dual deficiency of MyD88 and TRIF (Fig. 3C). Similarly, activation of EP4 reduced indomethacin-dependent increase in colonic RORγt^+^Foxp3^+^ T_regs_ in vehicle-treated, rather than antibiotic-treated, mice (Fig. 3C). These results suggest involvement of the commensal microbiota in the PGE_2_-dependent control of intestinal T_regs_.

### Alteration of the gut microbiota by PGE_2_-EP4 signaling

**Fig. 4:**
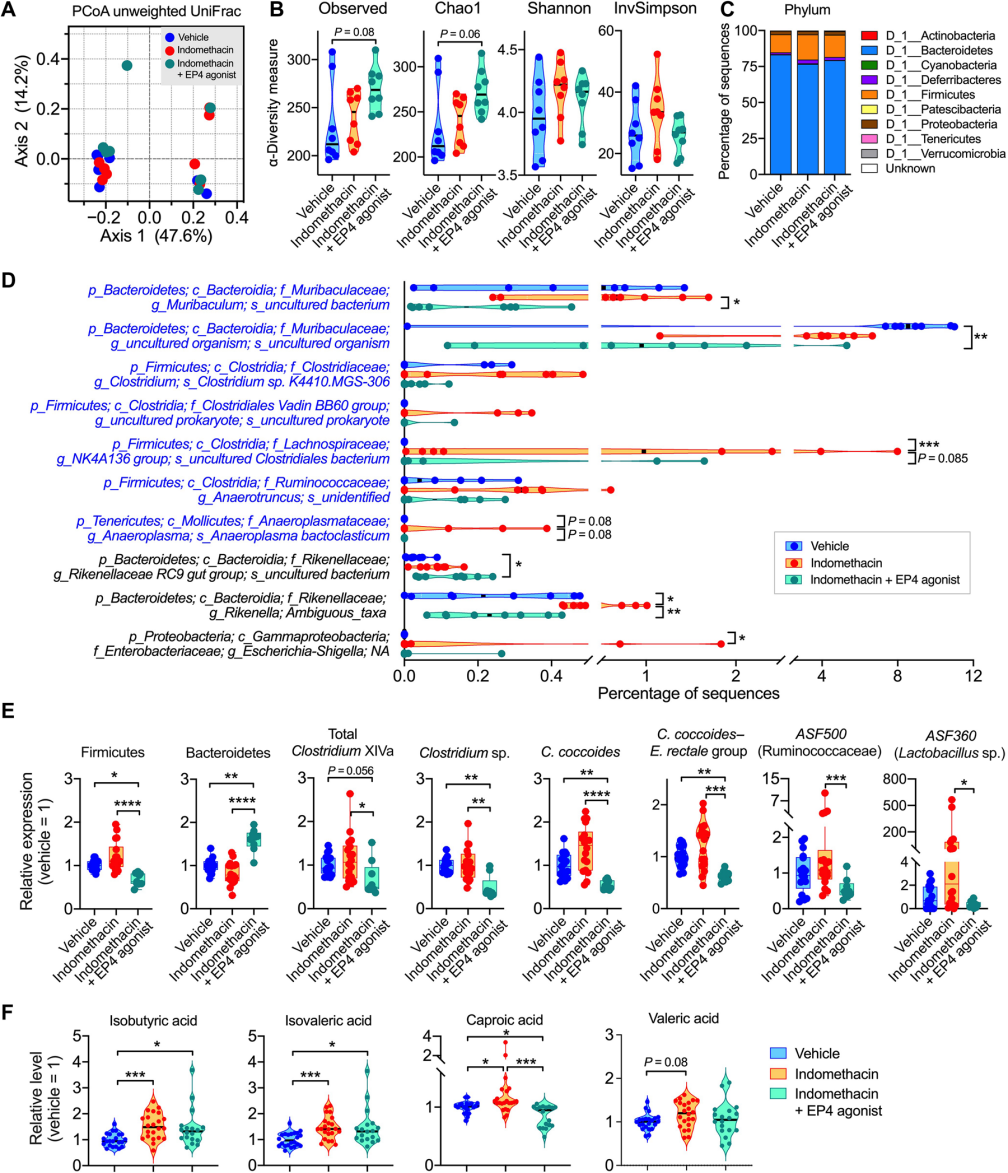
PGE_2_-EP4 signaling alters the gut microbiota. (**A** to **D**) DNA extracts of the cecal contents from C57BL/6 mice receiving vehicle control or indomethacin plus EP4 agonist for 5 days (*n* = 8 per group) were analyzed by 16*S* rRNA gene sequencing. (A) PCoA of cecal microbiota structure as measured by unweighted UniFrac distances among the three groups. (**B**) α-Diversity of the gut microbiome as measured by observed OTUs, Chao1 index, Shannon diversity, and InvSimpson index. (**C**) Phylum-level microbiota composition expressed as relative abundances. (**D**) Bacterial species changed in relative abundance among different groups. (**E**) 16*S* rRNA gene expression of indicated commensal microbiota in cecal contents obtained from C57BL/6 mice treated with vehicle (*n* = 17), indomethacin (*n* = 18), or indomethacin plus EP4 agonist L-902,688 (*n* = 9) for 5 days. Relative abundance of each group of bacteria to total cecal bacteria was determined by real-time quantitative polymerase chain reaction (qPCR) and normalized to the vehicle group. (**F**) Relative SCFA levels in cecal contents from mice treated with vehicle (*n* = 22), indomethacin (*n* = 22), or indomethacin plus EP4 agonist L-902,688 (*n* = 18). Amounts of SCFAs were detected using gas chromatography and normalized to the vehicle group. Each scatter dot plot represents data from one mouse (A, B, and D to F). Data plotted in box and whiskers bar graphs are pooled from five experiments and analyzed by nonparametric Kruskal-Wallis test with post hoc Dunn’s multiple comparisons test (E and F). *P < 0.05, **P < 0.01, ***P < 0.001, and ****P < 0.0001.

Use of NSAIDs has been reported to induce changes in the gut microbiota composition in humans and rodents (*33*–*35*). To examine whether PGE_2_-EP4 signaling modulates the gut microbiota, we collected cecal contents from mice that had been treated with indomethacin or that had been cotreated with indomethacin and an EP4 agonist and performed 16*S* ribosomal RNA (rRNA) gene metabarcoding to study microbiota composition. Principal components analysis (PCA) suggested that there were no differences in overall β-diversity of gut microbiota signatures measured by unweighted UniFrac distances among the three groups (Fig. 4A). The analysis of α-diversity indices (i.e., richness and evenness) showed that the three groups had also comparable observed operational taxonomic units (OTUs), Chao1 index, Shannon diversity, and Inverse Simpson (InvSimpson) indices (Fig. 4B). However, there were trends showing that the indomethacin^+^EP4 agonist–treated group had higher observed OTUs and Chao1 index (Fig. 4B).

We then asked whether PGE_2_-EP4 signaling alters specific bacterial communities. We found that treatment with indomethacin increased the abundance of the Firmicutes phylum and reduced the abundance of the Bacteroidetes phylum, and the changes in phylum-level microbiota composition by indomethacin were slightly reversed by cotreatment with the EP4 agonist (Fig. 4C). Indomethacin increased, but EP4 agonist reduced, several SCFA-producing bacteria belonging to the Muribaculaceae family or the *Clostridium* cluster XIVa (e.g., Lachnospiraceae and Ruminococcaceae) (Fig. 4D) (*5*). SCFA-producing bacteria such as Clostridia play critical roles in intestinal T_reg_ induction and accumulation. Furthermore, *Anaeroplasma bactoclasticum*, which promotes expression of immune-regulatory transforming growth factor–β in the gut (*36*), was also up-regulated by indomethacin and reduced by the EP4 agonist (Fig. 4D). To validate the 16*S* rRNA gene sequencing results, we used real-time quantitative polymerase chain reaction (qPCR) to detect gene expression of SCFA-producing bacteria in mice from independent cohorts. As confirmed, activation of EP4 significantly reduced the phylum Firmicutes and increased the phylum Bacteroidetes (Fig. 4E). In agreement with the 16*S* RNA sequencing (RNA-seq) results, EP4 activation notably decreased the amounts of Clostridia including total *Clostridium* cluster XIVa, *Clostridium* sp., *Clostridium coccoides*, *ASF500* (Ruminococcaceae), and *ASF360* (*Lactobacillus* sp.) (Fig. 4E). In addition, several aggressive microbial species such as species of the genera *Rikenella* and *Escherichia* were also increased by indomethacin but reduced by coadministration of EP4 agonist (Fig. 4D). *Rikenella* and *Escherichia* are pathogenic strains that can trigger mucosal inflammation and formation of mucus lesions (*37*). Therefore, besides SCFA-producing gut microbes, these pathogenic bacteria may also contribute to NSAID/PGE_2_-dependent modulation of gut epithelial injury and accumulation of T_regs_ in the intestine.

To examine whether PGE_2_ modulates SCFA production, we measured SCFA levels in cecal contents. Indomethacin treatment significantly increased the levels of caproic acids and branched SCFAs (isobutyric and isovaleric acids) in extracts of cecal contents (Fig. 4F). Indomethacin also had a trend to enhance valeric acid (Fig. 4F). Coadministration of EP4 agonist specifically reduced the levels of caproic acid (Fig. 4F). The levels of caproic and valeric acids were found significantly decreased in feces from patients with active CD compared to inactive CD or healthy controls (*38*, *39*). Clostridia are producers of caproic acid and branched SCFAs including isobutyric and isovaleric acids (*40*, *41*). Branched SCFAs, similar to conventional SCFAs modulate the host immune response (*42*). These corroboratory results thus suggest that PGE_2_-EP4 signaling reduces intestinal T_reg_ accumulation, at least partially, via reducing SCFA-producing microbiota.

### Modulation of mucosal T_regs_ and intestinal inflammation by EP4-modified gut microbiota

**Fig. 5:**
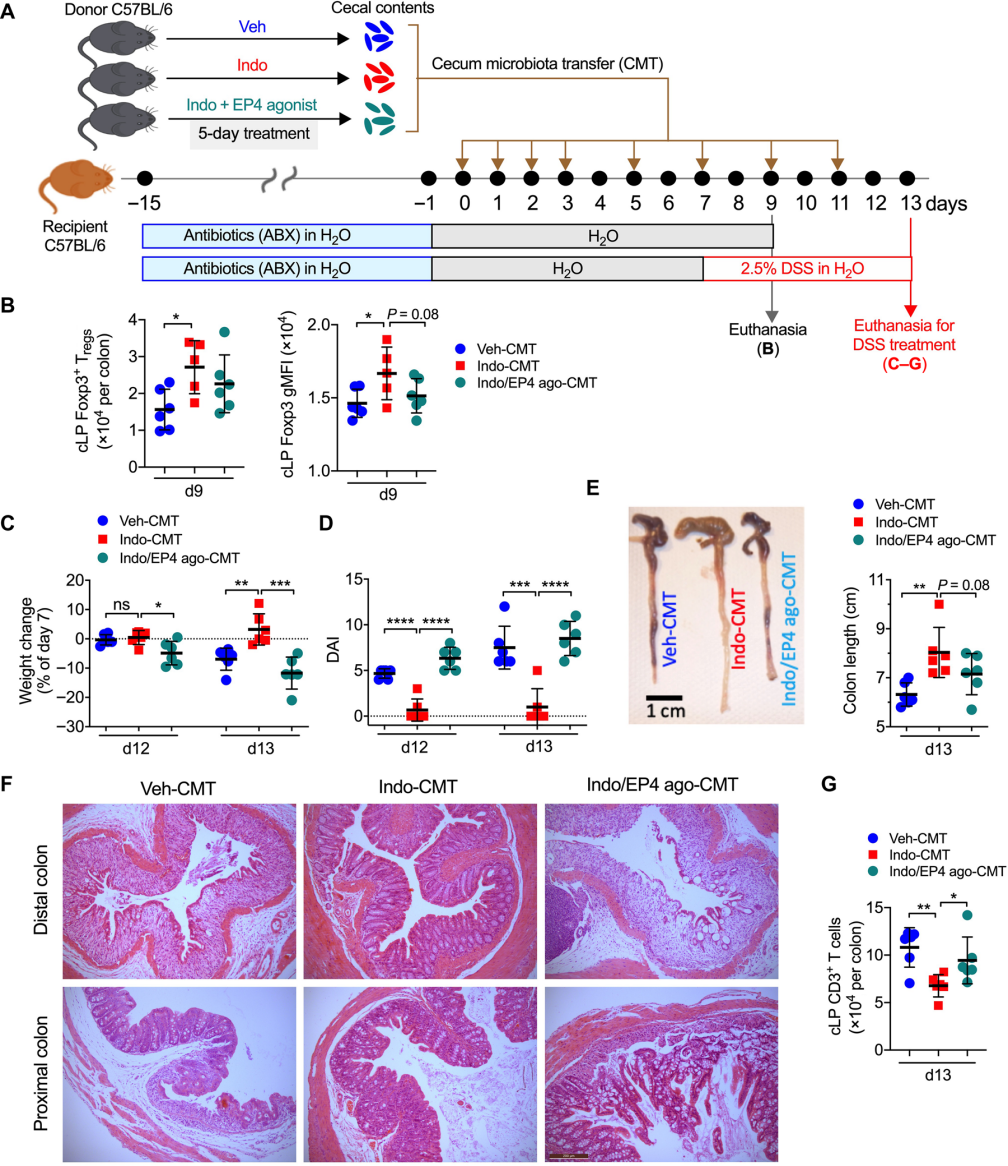
Altered gut microbiota by PGE_2_-EP4 signaling suppresses intestinal T_reg_ responses and promotes intestinal inflammation. (**A**) Experimental schematic diagram of cecal microbiota transplantation (CMT). Recipient C57BL/6 mice were pretreated with antibiotics for 2 weeks and rest for 1 day before receiving fresh cecal microbiota collected from C57BL/6 mice that had been treated with vehicle (Veh-CMT), indomethacin (Indo-CMT), or indomethacin plus EP4 agonist, L-902,688 (Indo/EP4 ago-CMT) for 5 days. After antibiotics treatment, some recipient mice were given back normal drinking water for 9 days before euthanized for analysis of colon immune cells [for (B)], while some other recipient mice were given back normal drinking water for 7 days, followed by administration with DSS in drinking water to induce colonic inflammation [for (C) to (G)]. (**B**) Numbers of colon LP Foxp3^+^ T_regs_ (left) and Foxp3 gMFI (right) in the recipient mice on day 9 after CMT (*n* = 5 to 6 each group). (**C**) Changes in body weight of recipient mice showing percentages of that at the beginning of DSS treatment (i.e., day 7). (**D**) DAI of the recipient mice (right) (*n* = 6 each group). (**E**) Representative images of the cecum and colon tissues (left) and colon lengths (right) of the recipient mice on day 13. Photo credit: Marie Goepp, University of Edinburgh. (**F**) Representative histological (hematoxylin and eosin–stained) images of distal (top row) and proximal (bottom row) colons of the recipient mice (all at same magnification, 100×). Scale bar, 200 μm. (**G**) Numbers of infiltrated CD3^+^ T cells in colon LP of the recipient mice. Each scatter dot plot in bar graphs represents data from one mouse. Data shown as means ± SD are analyzed by ANOVA with post hoc Holm-Sidak’s multiple comparisons test (B to E and G). *P < 0.05, **P < 0.01, ***P < 0.001, and ****P < 0.0001.

To investigate whether PGE_2_-modified microbiota modulates intestinal T_reg_ responses and inflammation, we adoptively transferred cecal microbiota obtained from WT C57BL/6 mice that had been treated with indomethacin, indomethacin plus EP4 agonist, or vehicle control into recipient WT C57BL/6 mice (Fig. 5A). Recipient mice were pretreated with antibiotics in drinking water for 2 weeks before receiving transplantation of microbiota and then received normal drinking water for another 10 days, followed by euthanasia to analyze colonic immune cell responses at the steady state. Some recipient mice received normal drinking water for 8 days after stopping antibiotic treatment, followed by receiving dextran sulfate sodium (DSS) in drinking water for an additional 6 days (Fig. 5A). Compared to mice that had received cecal microbiota from vehicle-treated mice, mice transplanted with cecal microbiota from indomethacin-treated mice had increased T_regs_ and Foxp3 expression at single-cell levels in the steady state (Fig. 5B). This increased expression was associated with prevention of body weight loss, reduced disease activity index (DAI), and increased colon length under DSS-induced inflammatory conditions in mice that had received cecal microbiota from indomethacin-treated mice (Fig. 5, C and D). In contrast, mice transplanted with cecal microbiota from mice that have been pretreated with indomethacin plus EP4 agonist had a trend to reduce colon T_regs_ and Foxp3 expression compared to mice received cecal microbiota from mice that have been pretreated with indomethacin (Fig. 5B). Furthermore, the severity of colitis in mice transplanted with cecal microbiota from mice that have been pretreated with both indomethacin and EP4 agonist was similar to mice that had received cecal microbiota from vehicle-treated mice but was significantly greater than that in mice received cecal microbiota from indomethacin-treated mice (Fig. 5, C to E). Histological analysis showed near normal proximal and distal colonic mucosa, or only scattered mild inflammatory changes, in mice transplanted with cecal microbiota from indomethacin-treated mice (Fig. 5F). There was widespread and variably severe mucosal ulceration with patches of almost complete loss of the crypt epithelium, with marked infiltration of fibrotic mucosal tissue by both acute and chronic inflammatory cells in mice that had received cecal microbiota from vehicle-treated mice, with more severe inflammatory and ulcerative changes in the distal colon compared with the proximal colon. In contrast, there was a less severe pattern of inflammation with more variable mild to moderate inflammatory cell infiltration with only patchy partial loss of mucosal crypt epithelium in those mice transplanted with cecal microbiota from indomethacin- and EP4 agonist–treated mice, again with greater inflammatory changes in the distal colon compared with the proximal colon (Fig. 5F). This was associated with reduced T cell infiltration to colon LP in mice that have received cecal microbiota from indomethacin-treated mice compared to the other two groups (Fig. 5G). Together, these results suggest that PGE_2_-EP4 signaling–modified gut microbiota contributes to inhibition of mucosal T_regs_ and exacerbation of intestinal inflammation.

### Increased colonic T_reg_ accumulation in MNP-specific EP4-deficient mice

**Fig. 6:**
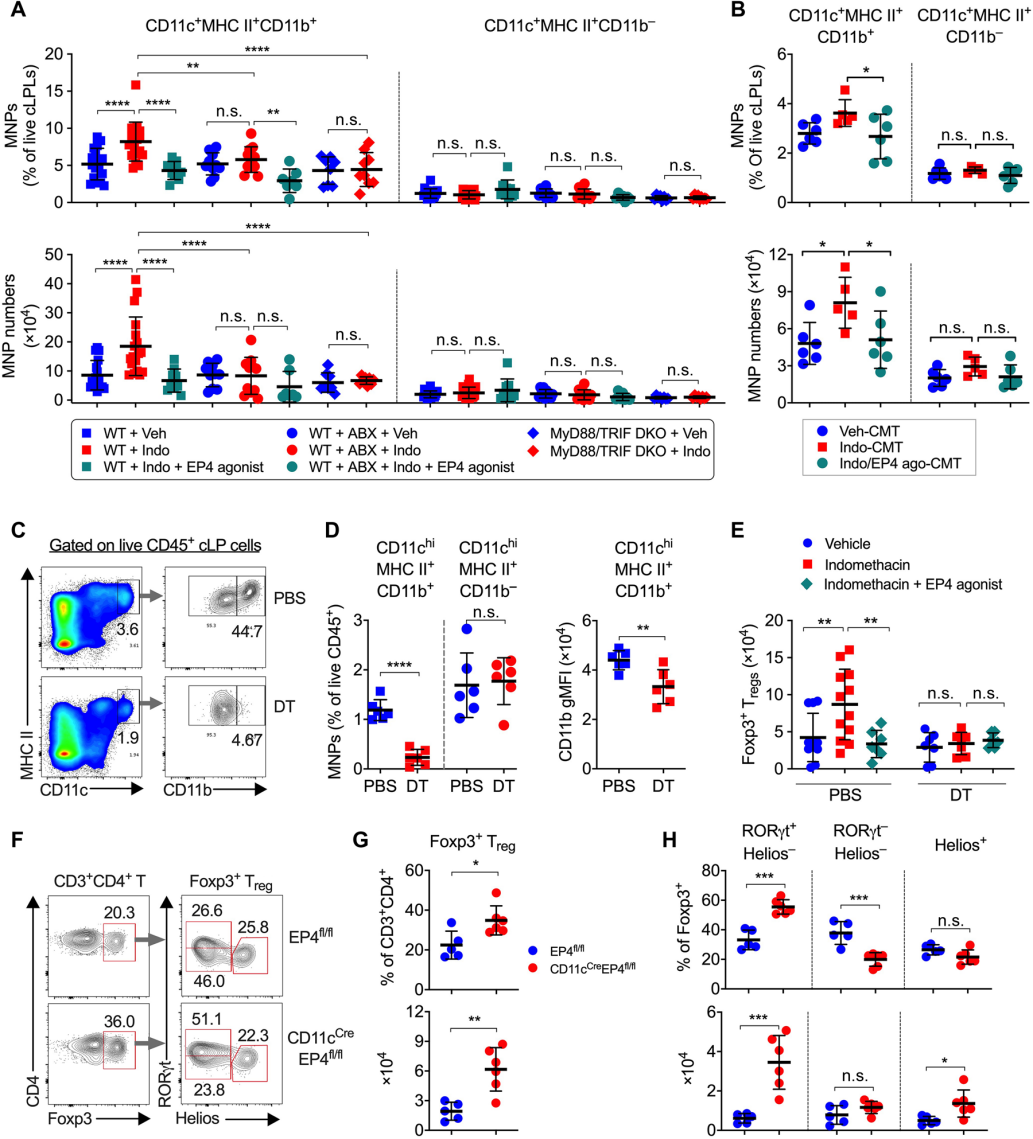
PGE_2_ inhibits intestinal MNPs via the gut microbiota, required for control of T_regs_. (**A**) Percentages (top) and numbers (bottom) of colon LP CD11c^+^MHC II^+^CD11b^+^ and CD11c^+^MHC II^+^CD11b^−^ MNPs from C57BL/6 WT and MyD88/TRIF double knockout mice treated with vehicle, indomethacin, or indomethacin plus EP4 agonist L-902,688 (*n* = 7 to 18). Antibiotics were administered to WT mice in drinking water for 2 weeks started 1 week before receiving indomethacin or vehicle. (**B**) Percentages (top) and numbers (bottom) of colon LP CD11c^+^MHC II^+^CD11b^+^ and CD11c^+^MHC II^+^CD11b^−^ MNPs in mice that were treated with antibiotics for 2 weeks, followed by transfer of cecal microbiota harvested from mice that had been administered with vehicle (Veh-CMT), indomethacin (Indo-CMT), or indomethacin plus EP4 agonist (Indo/EP4 ago-CMT) (*n* = 5 to 6). (**C** and **D**) Representative flow cytometry plots (C) and percentages (D) of colon LP CD11c^+^MHC II^+^CD11b^+^ and CD11c^+^MHC II^+^CD11b^−^ MNPs at colon LP of CD11b-DTR mice administered with diphtheria toxin (DT) or phosphate-buffered saline (PBS) (*n* = 6 each group). CD11b geometric gMFI of CD11c^+^MHC II^+^CD11b^+^ MNPs are also shown (D, right). (**E**) Numbers of colon LP CD45^+^CD3^+^CD4^+^Foxp3^+^ T_regs_ in CD11b-DTR mice treated with DT or PBS together with vehicle control, indomethacin, or indomethacin plus EP4 agonist (*n* = 6 to 12). (**F**) Representative flow cytometry plots of colonic Foxp3^+^ T_regs_ gated on live CD45^+^CD3^+^CD4^+^ T cells and RORγt^+^ or Helios^+^ T_regs_ in CD11c^Cre^EP4^fl/fl^ (*n* = 6) or control EP4^fl/fl^ mice (*n* = 5). (**G**) Percentage and numbers of Foxp3^+^ T_regs_. (**H**) Percentage and numbers of RORγt^+^Helios^−^, RORγt^−^Helios^−^or Helios^+^ T_regs_. Each scatter dot plot in bar graphs represents data from one mouse. Data shown as means ± SD are pooled from five (A), three (D and E), or one (B, G, and H) independent experiments and analyzed by ANOVA with post hoc Holm-Sidak’s multiple comparisons test (A, B, and E) or two-tailed unpaired Student’s *t* test (D, G, and H). *P < 0.05, **P < 0.01, ***P < 0.001, and ****P < 0.0001. n.s., not significant.

MNPs are critical to mediate the microbiota-dependent generation of intestinal Foxp3^+^ T_regs_ (*43*–*45*). To further study the interplay between PGE_2_ and the gut microbiota in the control of intestinal T_regs_, we examined MNPs in the colon. Comparing to treatment with vehicle control, administration of indomethacin increased both the frequency and number of colonic CD11c^+^MHC II^+^CD11b^+^ MNPs, and this was again inverted by coadministration of the EP4 agonist (Fig. 6A). CD11c^+^MHC II^+^CD11b^−^ MNPs were not affected by either indomethacin or EP4 activation (Fig. 6A). The effects of indomethacin and EP4 agonist on colonic CD11c^+^MHC II^+^CD11b^+^ MNPs were invisible in colons of antibiotic-treated mice or in MyD88/TRIF double-deficient mice (Fig. 6A). Moreover, transfer of gut microbiota from mice that had been treated with indomethacin increased colonic CD11c^+^MHC II^+^CD11b^+^ MNPs in host mice, and this was again reduced by transfer of gut microbiota from mice that had been cotreated with indomethacin and EP4 agonist (Fig. 6B). These results indicate that PGE_2_-EP4 signaling suppresses intestinal MNPs through modulating the gut microbiota.

Next, we asked whether CD11c^+^MHC II^+^CD11b^+^ MNPs mediate PGE_2_ suppression of intestinal T_regs_. To address this, we took advantage of mice that have CD11b promoter–driven expression of the human diphtheria toxin (DT) receptor (CD11b-DTR mice). Injection of DT selectively depleted colonic LP CD11c^+^MHC II^+^CD11b^+^ MNPs (by ~90%), rather than CD11c^+^MHC II^+^CD11b^−^ MNPs, and markedly reduced CD11b expression at the single-cell level in colonic CD11c^+^MHC II^+^CD11b^+^ MNPs (Fig. 6, C and D). While indomethacin increased and EP4 agonist decreased colonic Foxp3^+^ T_regs_ in phosphate-buffered saline (PBS)–treated CD11b-DTR mice, respectively, neither indomethacin nor EP4 agonist affected colonic T_regs_ in DT-treated CD11b-DTR mice (Fig. 6E). These results suggest that CD11c^+^MHC II^+^CD11b^+^ MNPs mediate PGE_2_ suppression of intestinal T_regs_. Furthermore, the C-C chemokine receptor type 2 (CCR2) mediates migration of monocyte-derived macrophages to the intestine. Mice deficient in CCR2 had reduced intestinal CD11c^+^MHC II^+^CD11b^+^ MNP numbers (by ~50%) and CD11b expression at the single-cell level compared to WT mice (fig. S4A). However, CCR2 deficiency did not prevent, if not enhanced, indomethacin-dependent increase in colonic Foxp3^+^ T_regs_ (fig. S4B), suggesting a role for gut resident but not monocyte-derived migratory MNPs in PGE_2_-dependent inhibition of intestinal T_regs_.

We further asked whether EP4 signaling in MNPs suppresses colonic T_regs_. To address this question, we crossed EP4-flox mice to CD11c-Cre mice to generate MNP-specific EP4-deficient mice. Inactivation of EP4 signaling in MNPs increased both percentages and absolute numbers of Foxp3^+^ T_regs_ in the colon (Fig. 6, F and G). Subsequent analysis revealed that EP4 deficiency in MNPs specifically boosted colonic RORγt^+^Helios^−^ T_regs_ and had few, if not inhibitory, effects on the RORγt^−^Helios^−^ T_reg_ subset (Fig. 6, F and H). Together, these results indicate that EP4 signaling in MNPs is responsible for PGE_2_ inhibition of colonic RORγt^+^ T_regs_.

### Impaired type I IFN signaling responsible for PGE_2_ suppression of intestinal MNPs and T_regs_

**Fig. 7:**
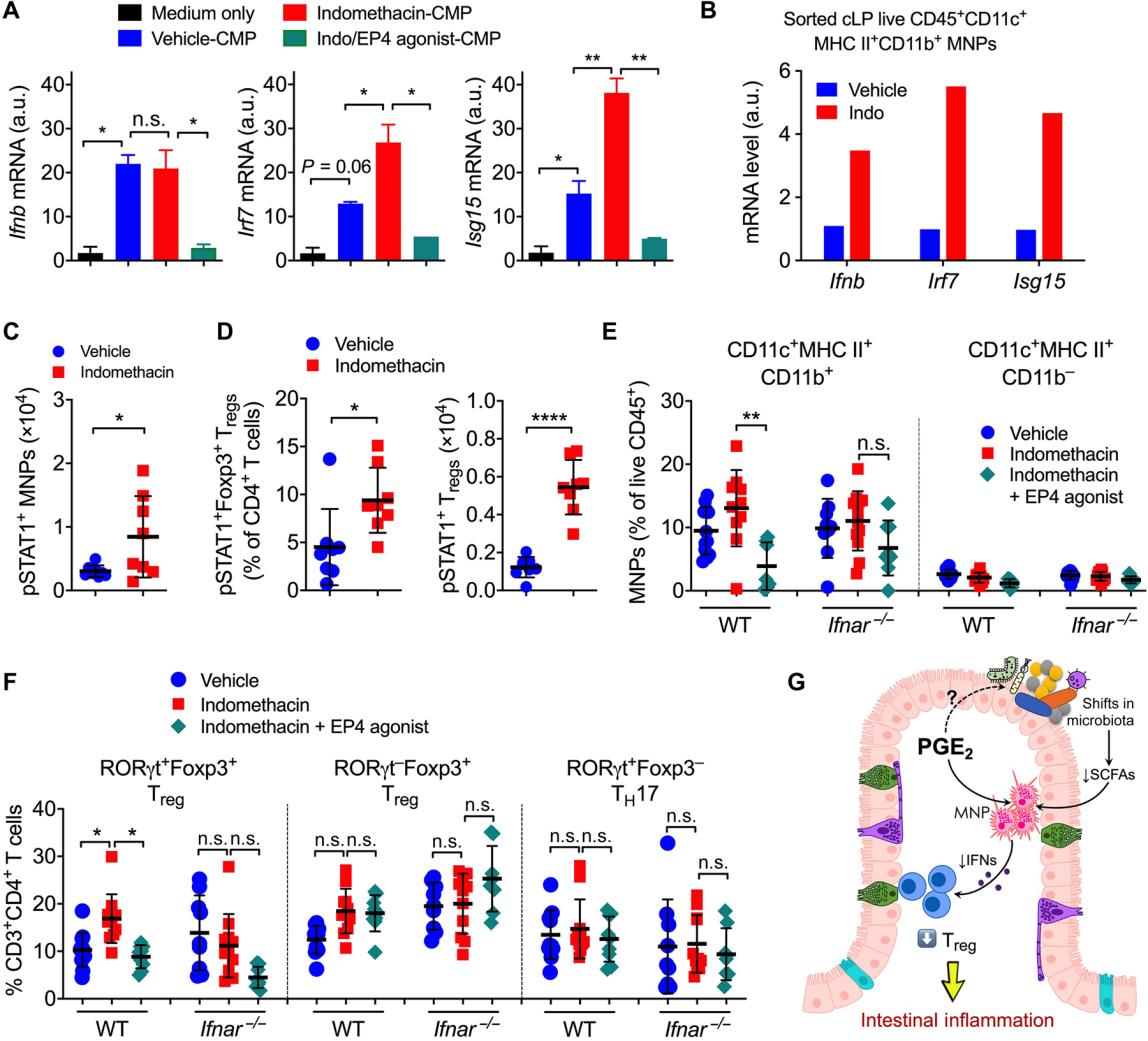
Type I IFNs produced by MNPs mediate PGE_2_ suppression of intestinal T_regs_. (**A**) Gene expression of *Ifnb*, *Irf7*, and *Isg15* in BMDCs cultured with CMPs that were isolated from mice treated with vehicle, indomethacin, or indomethacin plus EP4 agonist. Data shown as means ± SEM of duplicates are representative of two independent experiments. a.u., arbitrary units. (**B**) Gene expression of *Ifnb*, *Irf7*, and *Isg15* in sorted colonic MNPs from vehicle- or indomethacin-treated mice. Each sample was pooled from three to four mice. Data shown as mean gene expression of technical duplicates or triplicates are representative of two independent experiments. (**C**) Numbers of colonic phosphorylated STAT1–positive (pSTAT1^+^) MNPs and T_regs_ in mice treated with vehicle or indomethacin (*n* = 8 each). (**D**) Percentages and numbers of colon LP pSTAT1^+^Foxp3^+^ T_regs_. (**E**) Percentages of colon LP CD11c^+^MHC II^+^CD11b^+^ and CD11c^+^MHC II^+^CD11b^−^ MNPs in WT and *Ifnar^−/−^* mice treated with vehicle, indomethacin, or indomethacin plus EP4 agonist (*n* = 7 to 12). (**F**) Percentages of colon LP RORγt^+^Foxp3^+^ T_regs_, RORγt^−^Foxp3^+^ T_regs_, and RORγt^+^Foxp3^−^ T_H_17 cells. Each scatter dot plot represents data from one mouse. Data shown as means ± SD are pooled from two (C and D) or four (E and F) independent experiments. Two-tailed unpaired Student’s *t* test (C and D) or ANOVA with Holm-Sidak’s multiple comparisons test (A, E, and F). *P < 0.05, **P < 0.01, and ****P < 0.0001. n.s., not significant. (**G**) Diagram illustrating how PGE_2_ suppresses intestinal T_reg_ responses through shaping the gut microbiota composition and modulation of MNP functions.

MNPs mediate intestinal T_reg_ development and expansion by producing soluble mediators such as type I IFNs (*46*, *47*). The IFN-α/β receptor (IFNAR) signaling pathway has been shown to be required for T_reg_ development and function, especially under stress conditions or in a competitive environment (*48*). To test whether PGE_2_ regulates type I IFN signaling in intestinal CD11c^+^MHC II^+^CD11b^+^ MNPs, we cultured bone morrow–derived dendritic cells (BMDCs) without (medium only) or with cecal microbial products (CMPs; which might include pathogen- or microbe-associated molecular patterns including gut microbial metabolites) obtained from mice that had been treated with vehicle control, indomethacin, or indomethacin plus EP4 agonist. We then measured mRNA expression of type I IFN signaling pathway genes in BMDCs by real-time qPCR. BMDCs cultured with CMPs from control mice were characterized by higher expression of type I IFN (e.g., *Ifnb*) and the downstream genes (e.g., *Irf7* and *Isg15*) compared to that cultured with medium only (Fig. 7A). Expression of *Irf7* and *Isg15* was further increased in BMDCs cultured with CMPs obtained from mice that had been treated with indomethacin compared to that cultured with CMPs obtained from control mice (Fig. 7A). In contrast, mRNA expression levels of all these genes were markedly down-regulated in BMDCs that had been cultured with CMPs obtained from mice that have been cotreated with indomethacin and EP4 agonist (Fig. 7A). Moreover, colonic CD11c^+^MHC II^+^ MNPs sorted from indomethacin-treated mice expressed higher levels of type I IFN signaling pathway genes (e.g., *Ifnb*, *Irf7*, and *Isg15*) compared to MNPs sorted from colons of vehicle-treated mice (Fig. 7B). Colonic MNPs in mice treated with indomethacin had higher levels of phosphorylated signal transducers and activators of transcription 1 (STAT1) than that in vehicle-treated mice (Fig. 7C). These results suggest that modulation of gut microbial products by PGE_2_-EP4 signaling inhibits type I IFN production and signaling in MNPs. Similarly, colonic T_regs_ from indomethacin-treated mice also had more T_regs_ expressing phosphorylated STAT1 compared to vehicle-treated mice (Fig. 7D).

We next examined whether type I IFN signaling is required for PGE_2_ control of intestinal T_regs_ using IFNAR α chain (*Ifnar*)–deficient mice. Again, indomethacin increased colonic CD11c^+^MHC II^+^CD11b^+^ MNPs and RORγt^+^Foxp3^+^ T_regs_, and this was prevented by EP4 agonist in WT mice (Fig. 7 E and F). However, neither indomethacin nor EP4 agonist had effects on colonic CD11c^+^MHC II^+^CD11b^+^ MNPs and RORγt^+^Foxp3^+^ T_regs_ in *Ifnar*-deficient mice (Fig. 7, E and F). These results thus suggest that the PGE_2_-modified microbiota suppresses intestinal T_regs_ by down-regulating type I IFN signaling via MNPs.

## DISCUSSION

In this report, we have demonstrated that PGE_2_, the well-known mediator of inflammation, negatively controls intestinal T_reg_ responses. Endogenous PGE_2_-EP4 signaling alters the gut microbial community, e.g., reducing SCFA-producing bacteria, which in turn down-regulates MNP production of type I IFNs, leading to repression of intestinal T_reg_ accumulation and augmentation of intestinal inflammation (Fig. 7G). In contrast, blockade of PGE_2_ signaling (e.g., by NSAIDs) increases beneficial microbes and also some invasive microbes, likely as a consequence of damage to the epithelium, which augments MNP production of IFNs, resulting in expansion of mucosal T_regs_ and limitation of intestinal inflammation.

There are several potential mechanisms underpinning EP4-dependent inhibition of intestinal T_regs_ as PGE_2_ could target multiple enteric cell types including epithelial cells, T cells, and MNPs. Mice with EP4 deficiency in T cells had comparable numbers of intestinal T_regs_ to EP4-sufficient mice, excluding a role for EP4 signaling in T cells. Specific deletion of EP4 in epithelial cells (e.g., driven by Villin-cre) impaired colon homeostasis, increased epithelial cell apoptosis and immune cell infiltration, and exacerbated DSS-induced mucosal inflammation (*49*). Although T_reg_ accumulation in the colon in epithelial EP4–deficient mice has not been assessed, gene expression of Foxp3 that is exclusively expressed on T_regs_ in colon tissue is comparable between epithelial-specific EP4 deficient and control mice (*49*). Thus, endogenous PGE_2_ is unlikely to act through epithelial cells for T_reg_ inhibition in the naïve intestine. However, use of NSAID (e.g., indomethacin) may destroy the epithelium, leading to translocation of commensal microbes (*19*). Attachment of some invasive commensal microbes (e.g., *Helicobactor hepaticus*) to the epithelium is believed to favorably increase T_reg_ accumulation in the gut (*50*). Specific activation of EP4 agonism may thus contribute to reversing NSAID-dependent increase in intestinal T_regs_ by preventing NSAID-dependent epithelial injury. This is, at somewhat extend, supported by the finding that EP4 agonist alone (without indomethacin) had few effects on colonic T_reg_ accumulation (fig. S3). Therefore, a possibility that indomethacin increases colonic T_regs_, at least partly, through epithelial injury and attachment of the invasive microbes cannot be excluded.

Blockade of PGE_2_ biosynthesis by NSAIDs alters the gut microbiota by expanding the beneficial SCFA-producing microbes and increasing SCFA secretion in the intestine, which was reversed by the EP4 agonist. Given an increased ratio of Firmicutes to Bacteroidetes has been reported to be related to anti-inflammatory potential under various inflammatory conditions (*3*), our results showing that PGE_2_-EP4 signaling reduced Firmicutes but increased Bacteroidetes at the phylum level may have implications in modulation of intestinal inflammation. Our gut microbiota transfer experiments demonstrated that EP4-modified commensal microbes efficiently suppressed intestinal T_regs_ and mediated intestinal inflammation. Furthermore, our findings on the manipulation of gut microbiota in mice are in keeping with observations in humans showing that ingestion of antipyretic analgesics (i.e., NSAIDs or paracetamol that also can inhibit PG synthesis) increased the abundance of beneficial gut commensals such as Verrucomicrobia and SCFA-producing bacteria such as *Butyrivibrio* and Clostridiaceae (*35*). A recent report on in vitro culture of commensal bacteria suggested that most NSAIDs, including indomethacin and aspirin, were unlikely to affect bacterial growth on various microbial strains (*51*). PGE_2_ was reported to promote the growth of some bacterial strains such as *Escherichia coli* (*52*). Therefore, further studies are needed to decipher whether PGE_2_ acts directly on the gut microbiota or indirectly on host cells to regulate the growth, survival, or function of specific gut commensal bacterial strains.

Another possible mechanism for EP4 suppression of colonic T_regs_ is through a direct action on MNPs. Manipulation of PGE_2_-EP4 signaling (i.e., using COX inhibitors or EP4 agonist) alters the numbers of intestinal CD11c^+^CD11b^+^MHC II^+^ MNPs, and depletion of CD11b^+^ MNPs prevented PGE_2_-dependent regulation of intestinal T_regs_. CCR2-expressing monocyte-derived MNPs are critical for intestinal T_H_17 cell responses (*53*), but these migratory MNPs are unlikely to be involved in PGE_2_-dependent regulation of intestinal T_reg_ responses, indicating a role for gut resident MNPs. Notably, deletion of EP4 in CD11c^+^ MNPs increased accumulation of colonic T_regs_, especially the RORγt^+^ subset, indicating an essential role for EP4 signaling in MNPs. Thus, PGE_2_-modified gut microbiota controls MNP production of type I IFNs, which in turn mediates intestinal T_regs_ and MNPs themselves. This is consistent with a recent report showing that gut commensals stimulated CD11b^+^ dendritic cells to produce IFN-β, which augmented the proliferation of intestinal T_regs_ (*46*), although IFNAR signaling in T_regs_ was reported to impair T_reg_ suppressive function (*54*).

PGE_2_-EP4 signaling more specifically suppresses RORγt^+^ T_regs_ than the RORγt^−^ T_reg_ subpopulation. Comparing to RORγt^−^Foxp3^+^ T_regs_, RORγt^+^Foxp3^+^ T_regs_ express higher levels of mucosal resident T_reg_-associated genes such as *Ffar2* (*31*) that encodes the SCFA receptor G protein-coupled receptor 43 (GPR43). The SCFA-GPR43 axis is critical for the development, recruitment, and expansion of colonic T_regs_ (*10*). IFNAR-sufficient T_regs_ similarly express greater *Ffar2* and *Rorc* genes than IFNAR-deficient T_regs_ (*54*). Therefore, higher levels of SCFA and type I IFN in the intestine of indomethacin-treated mice may enhance the accumulation of GPR43-expressing RORγt^+^ T_regs_ compared to that in the mice that were treated with vehicle or indomethacin plus EP4 agonist. This may be, at least partially, responsible for a specific effect of the EP4-IFN axis on RORγt^+^ T_reg_ subpopulation.

Overactivation of the PGE_2_ pathway including PGE_2_ biosynthesis and its receptor signaling is an outstanding marker under most, if not all, human inflammatory conditions such as multiple sclerosis, rheumatoid arthritis, IBD, and cancers (*14*). Together with our previous findings (*26*, *27*), results from this current study imply PGE_2_ as a common inflammatory mediator for immune inflammation by balancing T cell responses, i.e., enhancing T_reg_ but limiting effector T cell responses. This is further supported by a recent study demonstrating that PGE_2_ exacerbates tumor necrosis factor (TNF)–induced inflammatory responses in human intestinal epithelial cells from patients with IBD who are resistant to TNF inhibitor therapy (*55*). Moreover, studies from multiple groups including ourselves have shown that lack of PGE_2_-EP4 signaling in T cells reduced both chemical-triggered acute and naïve T cell transfer–induced chronic intestinal inflammation, associated with reduction of inflammatory T_H_1 and/or T_H_17 cell responses (*27*, *56*). Thus, PGE_2_ mediates intestinal inflammation possibly through actions on both adaptive T cells and innate MNPs, and at least in the latter scenario, the gut microbiota is involved.

Collectively, we have defined a role for PGE_2_ in control of intestinal T_reg_ accumulation, which involves MNPs and the gut microbiota, leading to facilitation of mucosal inflammation. Our findings provide an explanation for human genetic findings of the association between *PTGER4* (EP4) gene polymorphisms and IBD susceptibility and suggest a potential therapeutic strategy for treating intestinal inflammation by targeting the PGE_2_-EP4-microbiota-MNP-T_reg_ cascade.

## MATERIALS AND METHODS

### Mice

*Rag1^−/−^*, MyD88^−/−^TRIF^−/−^, Ifnar^−/−^, CD11b-DTR, Ccr2^−/−^, Lck^Cre^EP4^fl/fl^, and WT C57BL/6 mice were bred and maintained under specific pathogen-free conditions in accredited animal facilities at the University of Edinburgh. To generate MNP-specific EP4-deficient mice, we crossed EP4-floxed mice to CD11c-Cre-GFP mice (JAX 007567). WT mice were bred in our own animal facilities or purchased from Harlan, UK. Age (>7 weeks old) and sex-matched mice were used. Mice were randomly allocated into different groups and analyzed individually. No mice were excluded from the analysis except exclusions due to technical errors in preparation of intestinal LP leukocytes. All experiments were conducted in accordance with the U.K. Animals (Scientific Procedures) Act of 1986 with local ethical approval from the University of Edinburgh Animal Welfare and Ethical Review Body.

### Cecal microbiota transplantation, colitis, and treatments with small molecular compounds

Recipient specific pathogen–free (SPF) WT C57BL/6 mice were pretreated with antibiotics for 2 weeks and rest for 1 day before receiving fresh cecal microbiota collected from SPF WT C57BL/6 mice that had been treated with vehicle, indomethacin (5 mg/kg per day in drinking water), or indomethacin plus EP4 agonist L-902,688 (10 μg per mouse per day via daily intraperitoneal injection) for 5 days. After antibiotics treatment, some recipient mice were given normal drinking water for another 9 days before euthanasia for analysis of colon immune cells in the steady state, while other recipient mice were administered with normal drinking water for 7 days, followed by 2.5% (w/v) of DSS (molecular weight, 36 to 50 kDa; MP Biochemicals) in drinking water for six consecutive days to induce colonic inflammation. The DAI was scored by the following system: body weight, 0 (no or <1% weight loss compared to day 0 body weight), 1 (1 to 5% weight loss), 2 (5 to 10% weight loss), 3 (10 to 20% weight loss), and 4 (>20% weight loss); bleeding, 0 (no bleeding), 1 (blood present in/on feces), 2 (visible blood in rectum), and 4 (visible blood on fur); stool consistency, 0 (well-formed/normal stool), 1 (pasty/semiformed stool), 2 (pasty stool with some blood), 3 (diarrhea that does not adhere to anus), and 4 (diarrhea that does adhere to anus); general appearance, 0 (normal), 1 (piloerection only), 2 (piloerection and lethargy), and 4 (atoxic, motionless and sunken eyes). Mice were immediately culled when body weight loss was greater than 25% or the total colitis score is 12 or higher. Indomethacin (5 mg/kg per day or indicated doses) and vehicle (0.5% EtOH) were administrated through drinking water that was refreshed every 2 to 3 days. Butaprost (10 μg per injection; Abcam), L-902,688 (10 μg per injection; Cayman Chemical), and control (0.5% EtOH in PBS) were used by daily intraperitoneal injections. Antibiotics (containing ampicillin, gentamycin, metronidazole, neomycin, and vancomycin, each 0.5 mg/ml) and sucralose (4 mg/ml) were used in drinking water. DT (25 ng/g of bogy weight; Sigma-Aldrich) was injected intraperitoneally every 48 hours.

### Histology

Intestine samples were fixed with 10% neutral buffered formalin solution (Sigma-Aldrich) and embedded in paraffin, and 5-μm sections were used for staining with hematoxylin and eosin.

### Isolation and staining of intestinal LP leukocytes

Intestinal LP cells were isolated as described previously (*19*). For surface staining, cells were first stained with the Fixable Viability Dye eFluor 780 (eBioscience) on ice for 30 min. After wash, cells were stained on ice for another 30 min with anti-CD45 (clone 30-F11), anti-CD3e (clone 145-2C11), anti-CD4 (clone GK1.5), anti-CD25 (clone PC61.5), anti-CD11c (clone N418), anti-CD11b (clone M1/70), anti-B220 (clone RA3-6B2), and anti-mouse I-A/I-E antibody (clone M5/114.15.2). For staining of transcription factors, cells were fixed in the Foxp3/Transcription Factor Fix Buffer (eBioscience) for 2 hours or overnight, followed by staining with anti-mouse Foxp3 (clone FJK-16s), anti-mouse RORγt (clone B2D), anti-mouse Helios (clone 22F6), and mouse anti-Stat1 (pY701) (clone 4a) for at least 1 hour. All antibodies were purchased from eBioscience, BioLegend, or BD Biosciences. Flow cytometry was performed on the BD LSRFortessa (BD Biosciences) and analyzed by FlowJo software (Tree Star).

### BMDC differentiation and stimulation

Bone marrow cells from femurs were cultured at 4 × 10^5^ cells/ml of culture medium with granulocyte-macrophage colony-stimulating factor (GM-CSF) (20 ng/ml) in complete RPMI 1640 to induce the differentiation of BMDCs. RPMI 1640 with GM-CSF was refreshed every 3 days. On day 9, BMDCs were harvested by collecting the nonadherent cells after gently swirling the plate and restimulated for 6 hours with RPMI 1640 alone or cecal microbial products (CMPs) obtained from mice that received various treatments as indicated in related figure legends. CMPs were prepared by dissolving cecal contents with sterile PBS (100 mg/ml) with vigorous vertex followed by centrifugation at 10,000 rpm for 2 min and filtered through a 0.22-μm Millex-GP filter unit. The CMP solution was then stored at −20°C and diluted by 40 times using PBS(−) for use in experiments. Cells were cultured at 37°C with 5% CO_2_.

### Oxylipin analysis

Small intestine and colon samples were weighed and homogenized with ceramic beads in 1 ml of antioxidation buffer containing 100 μM diethylenetriamine pentaacetic acid and 100 μM butylated hydroxytoluene in PBS using a Bead Ruptor Elite for 2 × 30 s intervals at 6 m/s, under cooled nitrogen gas (4°C). Samples were spiked with 2.1 to 2.9 ng of PGE_2_-d_4_, PGD_2_-d_4_, PGF_2_α-d_4_, and TXB_2_-d_4_ standards (Cayman Chemical) before homogenization. Lipids were extracted by adding a 1.25-ml solvent mixture (1 M acetic acid/isopropanol/hexane; 2:20:30, v/v/v) to 0.5-ml supernatants in a glass extraction vial and vortexed for 30 s. A total of 1.25 ml of hexane was added to samples, and after vortexing for 30 s, tubes were centrifuged (500*g* for 5 min at 4°C) to recover lipids in the upper hexane layer (aqueous phase), which was transferred to a clean tube. Aqueous samples were reextracted as above by addition of 1.25 ml of hexane, and upper layers were combined. Lipid extraction from the lower aqueous layer was then completed according to the Bligh and Dyer technique using sequential additions of methanol, chloroform, and water, and the lower layer was recovered following centrifugation as above and combined with the upper layers from the first stage of extraction. Solvent was dried under vacuum, and lipid extract was reconstituted in 200 μl of high-performance liquid chromatography grade methanol. Lipids were separated by liquid chromatography using a gradient of 30 to 100% B over 20 min (A, water:Mob B 95:5 + 0.1% acetic acid; B, acetonitrile:methanol –80:15 + 0.1% acetic acid) on an Eclipse Plus C18 Column (Agilent) and analyzed on a Sciex QTRAP 6500 liquid chromatography–tandem mass spectrometry system. Source conditions are as follows: temperature at 475°C, IS-4500, GS1 60, GS2 60, and CUR 35. Lipids were detecting using multiple reaction monitoring with the following parent to daughter ion transitions: PGD_1_ and PGE_1_ [M-H], 353.2/317.2; PGD_2_, 8-iso PGE_2_, and PGE_2_ [M-H], 351.2/271.1; PGF_2α_ [M-H], 353.2/309.2; 6-keto PGF_1α_ [M-H], 369.2/163.1; TXB_2_ [M-H], 369.2/169.1; 13,14-dihydro-15-keto-PGE_2_ [M-H], 351.2/235.1. Deuterated internal standards were monitored using parent-to-daughter ion transitions of the following: TXB_2_-d_4_ [M-H], 373.2/173.1; PGE_2_-d_4_ and PGD_2_-d_4_ [M-H], 355.2/275.1; PGF_2α_-d_4_ [M-H], 357.5/313.2. Chromatographic peaks were integrated using MultiQuant 3.0.2 software (Sciex). Peaks were only selected when their intensity exceeded three times above the baseline noise. The ratio of analyte peak areas to internal standard was taken, and lipids were quantified using a standard curve made up and run at the same time as the samples. Each oxylipin was then standardized per milligram of colon tissue.

### SCFA profiling

The levels of SCFA (acetic acid, propionic acid, butyric acid, valeric acid, caproic acid, heptanoic acid, and caprylic acid) and branched SCFA (isobutyric acid and isovaleric acid) in cecal contents were detected as described previously (*57*). Briefly, the SCFA and branched SCFA (isobutyric acid and isovaleric acid) were extracted from acidified slurries three times in total using diethyl ether. Extracts were analyzed using gas chromatography (Agilent 7890A) with flame ionization detector, as described previously (cite paper above). Each of the SCFA was quantified against calibration curves plotted using authentic external standards [acetic acid (174.8 mM), propionic acid (133.4 mM), butyric acid (107.3 mM), valeric acid (89.2 mM), caproic acid (77.4 mM), heptanoic acid (69.8 mM), caprylic acid (58.5 mM), isobutyric acid (106.5 mM), and isovaleric acid (86.9 mM) all stored in 2 M NaOH and using 2-ethylbutyric acid (73.2 mM) as internal standard. Concentration of SCFA in cecal contents were calculated as micromoles per gram and normalized to the vehicle control group.

### Real-time PCR

RNA purification from sorted MNPs was performed using the RNeasy Mini Kit (QIAGEN). Complementary DNA (cDNA) was obtained by reverse transcription using the High-Capacity cDNA Reverse Transcription Kits (ThermoFisher Scientific). Samples were analyzed by real-time PCR with GoTaq qPCR Master Mix (Promega) on the Applied Biosystems 7900HT Fast machine. Primers used are as follows: *Ifna*, 5′-GGACTTTGGATTCCCGCAGGAGAAG-3′ (forward) and 5′-GCTGCATCAGACAGCCTTGCAGGTC-3′ (reverse); *Ifnb*, 5′-AACCTCACCTACAGGGCGGACTTCA-3′ (forward) and 5′-TCCCACGTCAATCTTTCCTCTTGCTTT-3′ (reverse); *Irf7*, 5′-CCCCATCTTCGACTTCAGAG-3′ (forward) and 5′-AAGGAAGCACTCGATGTCGT-3′ (reverse); *Isg15*, 5′-TGACTGTGAGAGCAAGCAGC-3′ (forward) and 5′-CCCCAGCATCTTCACCTTTA-3′ (reverse); glyceraldehyde-3-phosphate dehydrogenase (*Gapdh*), 5′-TGAACGGGAAGCTCACTGG-3′ (forward) and 5′-TCCACCACCCTGTTGCTGTA-3′ (reverse). Expression was normalized to *Gapdh* and presented as relative expression to the control group by the 2^–ΔΔ*C*t^ method.

Total bacterial DNA was extracted from cecal contents using the QIAamp DNA Stool Mini Kit (QIAGEN) according to the manufacturer’s protocol. DNA concentration and quality in the extracts were determined by NanoDrop 1000 spectrophotometer (Thermo Fisher Scientific). Bacterial groups in cecal samples were measured by real-time PCR using GoTaq qPCR Master Mix (Promega) on Applied Biosystems 7900HT Fast or StepOne Plus Real-Time PCR Systems. Primers used for 16*S* rRNA gene qPCR are as follows (*58*–*63*): Firmicutes, 5′-GGAGYATGTGGTTTAATTCGAAGCA-3′ (forward; Firm934F) and 5′-AGCTGACGACAACCATGCAC-3′ (reverse; Firm1060R); Bacteroidetes, 5′-GGARCATGTGGTTTAATTCGATGAT-3′ (forward; Bact934F) and 5′-AGCTGACGACAACCATGCAG-3′ (reverse; Bact1060R); *Clostridium XIVa*, 5′-AAATGACGGTACCTGACTAA-3′ (forward) and 5′-CTTTGAGTTTCATTCTTGCGAA-3′ (reverse); *Clostridium* sp., 5′-CACCAAGGCGACGATCAGT-3′ (forward) and 5′-GAGTTTGGGCCGTGTCTCA-3′ (reverse); *C. coccoides* subgroup, 5′-AAATGACGGTACCTGACTAA-3′ (forward) and *C. coccoides* subgroup, 5′-CTTTGAGTTTCATTCTTGCGAA-3′ (reverse); *C. coccoides–Eubacteria rectale* group, 5′-CGGTACCTGACTAAGAAGC-3′ (forward) and 5′-AGTTTYATTCTTGCGAACG-3′ (reverse); *ASF500*, 5′-GTCGCATGGCACTGGACATC-3′ (forward; 500-183F) and 5′-CCTCAGGTACCGTCACTTGCTTC-3′ (reverse; 500-445R); *ASF360*, 5′-CTTCGGTGATGACGCTGG-3′ (forward; 360-81F) and 5′-GCAATAGCCATGCAGCTATTGTTG-3′ (reverse; 360-189R); all bacteria, 5′-TCCTACGGGAGGCAGCAGT-3′ (forward; UnivF) and 5′-GACTACCAGGGTATCTAATCCTGTT-3′ (reverse; UnivR). Expression was normalized to all bacterial DNA, and relative expression levels were calculated relatively to the vehicle control group by the 2^–ΔΔ*C*t^ method.

### 16*S* rRNA gene sequencing

Aliquots for sequencing of the 16*S* rRNA gene were first amplified with the V3-V4 region primers 341F (5′-CCTACGGGAGGCAGCAG-3′) and 518R (5′-ATTACCGCGGCTGCTGG-3′) as described previously (*64*). A reagent-only control (DNA extraction kit blank) and a mock bacterial community (HM-782D, BEI Resources, American Type Culture Collection, Manassas, VA) were also prepared in the same manner. A single library pool was compiled using equimolar concentrations of DNA as measured using a fluorometric assay (Qubit dsDNA Broad-Range Assay Kit, Invitrogen, UK). The Illumina MiSeq platform (Illumina, CA) was used for sequencing (Edinburgh Genomics, UK), using V2 chemistry and producing 250–base pair paired-end reads. Using the mock bacterial community data, the sequencing error rate was calculated as 0.01%. The raw sequence reads, with primers removed, are publicly available via the National Center for Biotechnology Information Sequence Read Archive under accession number PRJNA564944.

### 16*S* rRNA gene sequencing data analysis

For the raw 16*S* RNA data in FASTQ format, amplicon primers were first removed to prevent false-positive detection of chimeras using the cutadapt plugin (*65*). Ten thousand sequences were sampled at random, and the qualities at each base position were examined for determining the parameters of the denoising process. The paired-end reads were further trimmed, filtered, denoised, and merged using the DADA2 plugin (*66*) through the Wales supercomputer portal. A naïve Bayes classifier within QIIME2 v2019.10 (*67*) was trained against the Silva v132 database (https://arb-silva.de/) on the amplified region. In addition, a machine learning Python library scikit-learn (*68*) was used to classify OTUs on the basis of 100% sequence identity. A total of seven taxonomic levels were used for 16*S* rRNA datasets. For measuring diversity, we generated de novo phylogenetic trees through multiple sequence alignment, masking, tree building, and rooting using the multiple alignment using fast Fourier transform (MAFFT) program (*69*). Percent abundance of taxa was determined by calculating it as a proportion of the total read count across all samples. For instance, the percent abundance of the *i*th taxa in the *j*th sample is computed as

$$f(x_{i,j})=\frac{R_{i,j}}{\sum_{j=1}^{n}R_{i}}\times 100$$

where *n* is the total number of samples and *R* represents read counts of each taxa.

The OTU table was rarefied across samples to the 90% of the lowest sample depth with a random seed set as 123 to eliminate the bias caused by the different sample sizes. For the overall bacterial community within samples, α-diversity estimators including the observed species, the Chao1 index, the Shannon diversity, and the InvSimpson index were calculated using phyloseq (*70*). The α-diversity estimates were compared between three groups using nonparametric Kruskal-Wallis test along with Dunn’s multiple comparison correction. β-Diversity between samples was computed using the unweighted UniFrac distances (*71*).

### Statistical analysis

All data were expressed as means ± SD except that in Fig. 7A, where the data were expressed as means ± SEM as indicated in the figure legends. Statistical significance between two groups was examined by unpaired Student’s *t* test, while the analysis of variance (ANOVA) with post hoc Holm-Sidak’s multiple comparisons test was used to evaluate multiple groups. Statistical work was performed using Prism 8 software (GraphPad), and *P* < 0.05 was considered as significance.

## SUPPLEMENTARY MATERIALS

Supplementary material for this article is available at http://advances.sciencemag.org/cgi/content/full/7/7/eabd7954/DC1

## Appendix

View/request a protocol for this paper from *Bio-protocol*.

## References

[1] H. H. Uhlig, F. Powrie, Translating immunology into therapeutic concepts for inflammatory bowel disease. Annu. Rev. Immunol. 36, 755–781 (2018). 29677472 10.1146/annurev-immunol-042617-053055

[2] A. Basson, A. Trotter, A. Rodriguez-Palacios, F. Cominelli, Mucosal interactions between genetics, diet, and microbiome in inflammatory bowel disease. Front. Immunol. 7, (2016). 10.3389/fimmu.2016.00290 PMC4970383 27531998

[3] S. V. Lynch, O. Pedersen, The human intestinal microbiome in health and disease. N. Engl. J. Med. 375, 2369–2379 (2016). 27974040 10.1056/NEJMra1600266

[4] K. Honda, D. R. Littman, The microbiota in adaptive immune homeostasis and disease. Nature 535, 75–84 (2016). 27383982 10.1038/nature18848

[5] K. Atarashi, T. Tanoue, K. Oshima, W. Suda, Y. Nagano, H. Nishikawa, S. Fukuda, T. Saito, S. Narushima, K. Hase, S. Kim, J. V. Fritz, P. Wilmes, S. Ueha, K. Matsushima, H. Ohno, B. Olle, S. Sakaguchi, T. Taniguchi, H. Morita, M. Hattori, K. Honda, T_reg_ induction by a rationally selected mixture of Clostridia strains from the human microbiota. Nature 500, 232–236 (2013). 23842501 10.1038/nature12331

[6] C. Ohnmacht, J.-H. Park, S. Cording, J. B. Wing, K. Atarashi, Y. Obata, V. Gaboriau-Routhiau, R. Marques, S. Dulauroy, M. Fedoseeva, M. Busslinger, N. Cerf-Bensussan, I. G. Boneca, D. Voehringer, K. Hase, K. Honda, S. Sakaguchi, G. Eberl, The microbiota regulates type 2 immunity through RORγt^+^ T cells. Science 349, 989–993 (2015). 26160380 10.1126/science.aac4263

[7] E. Sefik, N. Geva-Zatorsky, S. Oh, L. Konnikova, D. Zemmour, A. M. McGuire, D. Burzyn, A. Ortiz-Lopez, M. Lobera, J. Yang, S. Ghosh, A. Earl, S. B. Snapper, R. Jupp, D. Kasper, D. Mathis, C. Benoist, Individual intestinal symbionts induce a distinct population of RORγ^+^ regulatory T cells. Science 349, 993–997 (2015). 26272906 10.1126/science.aaa9420 PMC4700932

[8] T. Tanoue, K. Atarashi, K. Honda, Development and maintenance of intestinal regulatory T cells. Nat. Rev. Immunol. 16, 295–309 (2016). 27087661 10.1038/nri.2016.36

[9] K. J. Maloy, F. Powrie, Intestinal homeostasis and its breakdown in inflammatory bowel disease. Nature 474, 298–306 (2011). 21677746 10.1038/nature10208

[10] P. M. Smith, M. R. Howitt, N. Panikov, M. Michaud, C. A. Gallini, M. Bohlooly-Y, J. N. Glickman, W. S. Garrett, The microbial metabolites, short-chain fatty acids, regulate colonic T_reg_ cell homeostasis. Science 341, 569–573 (2013). 23828891 10.1126/science.1241165 PMC3807819

[11] Y. Furusawa, Y. Obata, S. Fukuda, T. A. Endo, G. Nakato, D. Takahashi, Y. Nakanishi, C. Uetake, K. Kato, T. Kato, M. Takahashi, N. N. Fukuda, S. Murakami, E. Miyauchi, S. Hino, K. Atarashi, S. Onawa, Y. Fujimura, T. Lockett, J. M. Clarke, D. L. Topping, M. Tomita, S. Hori, O. Ohara, T. Morita, H. Koseki, J. Kikuchi, K. Honda, K. Hase, H. Ohno, Commensal microbe-derived butyrate induces the differentiation of colonic regulatory T cells. Nature 504, 446–450 (2013). 24226770 10.1038/nature12721

[12] N. Arpaia, C. Campbell, X. Fan, S. Dikiy, J. van der Veeken, P. deRoos, H. Liu, J. R. Cross, K. Pfeffer, P. J. Coffer, A. Y. Rudensky, Metabolites produced by commensal bacteria promote peripheral regulatory T-cell generation. Nature 504, 451–455 (2013). 24226773 10.1038/nature12726 PMC3869884

[13] C. Schiering, T. Krausgruber, A. Chomka, A. Fröhlich, K. Adelmann, E. A. Wohlfert, J. Pott, T. Griseri, J. Bollrath, A. N. Hegazy, O. J. Harrison, B. M. J. Owens, M. Löhning, Y. Belkaid, P. G. Fallon, F. Powrie, The alarmin IL-33 promotes regulatory T-cell function in the intestine. Nature 513, 564–568 (2014). 25043027 10.1038/nature13577 PMC4339042

[14] C. Yao, S. Narumiya, Prostaglandin-cytokine crosstalk in chronic inflammation. Br. J. Pharmacol. 176, 337–354 (2019). 30381825 10.1111/bph.14530 PMC6329627

[15] S. S. Thomas, K. W. Makar, L. Li, Y. Zheng, P. Yang, L. Levy, R. Y. Rudolph, P. D. Lampe, M. Yan, S. D. Markowitz, J. Bigler, J. W. Lampe, J. D. Potter, Tissue-specific patterns of gene expression in the epithelium and stroma of normal colon in healthy individuals in an aspirin intervention trial. BMC Med. Genet. 16, 18 (2015). 25927723 10.1186/s12881-015-0161-6 PMC4422425

[16] O. Ahrenstedt, R. Hällgren, L. Knutson, Jejunal release of prostaglandin E2 in Crohn’s disease: Relation to disease activity and first-degree relatives. J. Gastroenterol. Hepatol. 9, 539–543 (1994). 7865710 10.1111/j.1440-1746.1994.tb01557.x

[17] R. O. Day, G. G. Graham, Non-steroidal anti-inflammatory drugs (NSAIDs). BMJ 346, f3195 (2013). 23757736 10.1136/bmj.f3195

[18] K. Kabashima, T. Saji, T. Murata, M. Nagamachi, T. Matsuoka, E. Segi, K. Tsuboi, Y. Sugimoto, T. Kobayashi, Y. Miyachi, A. Ichikawa, S. Narumiya, The prostaglandin receptor EP4 suppresses colitis, mucosal damage and CD4 cell activation in the gut. J. Clin. Invest. 109, 883–893 (2002). 11927615 10.1172/JCI14459 PMC150928

[19] R. Duffin, R. A. O’Connor, S. Crittenden, T. Forster, C. Yu, X. Zheng, D. Smyth, C. T. Robb, F. Rossi, C. Skouras, S. Tang, J. Richards, A. Pellicoro, R. B. Weller, R. M. Breyer, D. J. Mole, J. P. Iredale, S. M. Anderton, S. Narumiya, R. M. Maizels, P. Ghazal, S. E. Howie, A. G. Rossi, C. Yao, Prostaglandin E_2_ constrains systemic inflammation through an innate lymphoid cell-IL-22 axis. Science 351, 1333–1338 (2016). 26989254 10.1126/science.aad9903 PMC4841390

[20] M. Roulis, C. Nikolaou, E. Kotsaki, E. Kaffe, N. Karagianni, V. Koliaraki, K. Salpea, J. Ragoussis, V. Aidinis, E. Martini, C. Becker, H. R. Herschman, S. Vetrano, S. Danese, G. Kollias, Intestinal myofibroblast-specific Tpl2-Cox-2-PGE_2_ pathway links innate sensing to epithelial homeostasis. Proc. Natl. Acad. Sci. U.S.A. 111, E4658–E4667 (2014). 25316791 10.1073/pnas.1415762111 PMC4217397

[21] Y. Zhang, A. Desai, S. Y. Yang, K. B. Bae, M. I. Antczak, S. P. Fink, S. Tiwari, J. E. Willis, N. S. Williams, D. M. Dawson, D. Wald, W.-D. Chen, Z. Wang, L. Kasturi, G. A. Larusch, L. He, F. Cominelli, L. Di Martino, Z. Djuric, G. L. Milne, M. Chance, J. Sanabria, C. Dealwis, D. Mikkola, J. Naidoo, S. Wei, H.-H. Tai, S. L. Gerson, J. M. Ready, B. Posner, J. K. V. Willson, S. D. Markowitz, Inhibition of the prostaglandin-degrading enzyme 15-PGDH potentiates tissue regeneration. Science 348, aaa2340 (2015). 26068857 10.1126/science.aaa2340 PMC4481126

[22] C. Libioulle, E. Louis, S. Hansoul, C. Sandor, F. Farnir, D. Franchimont, S. Vermeire, O. Dewit, M. de Vos, A. Dixon, B. Demarche, I. Gut, S. Heath, M. Foglio, L. Liang, D. Laukens, M. Mni, D. Zelenika, A. Van Gossum, P. Rutgeerts, J. Belaiche, M. Lathrop, M. Georges, Novel Crohn disease locus identified by genome-wide association maps to a gene desert on 5p13.1 and modulates expression of *PTGER4*. PLOS Genet. 3, e58 (2007). 17447842 10.1371/journal.pgen.0030058 PMC1853118

[23] J. Glas, J. Seiderer, D. Czamara, G. Pasciuto, J. Diegelmann, M. Wetzke, T. Olszak, C. Wolf, B. Müller-Myhsok, T. Balschun, J.-P. Achkar, M. I. Kamboh, A. Franke, R. H. Duerr, S. Brand, PTGER4 expression-modulating polymorphisms in the 5p13.1 region predispose to Crohn’s disease and affect NF-κB and XBP1 binding sites. PLOS ONE 7, e52873 (2012). 23300802 10.1371/journal.pone.0052873 PMC3531335

[24] J. C. Barrett, S. Hansoul, D. L. Nicolae, J. H. Cho, R. H. Duerr, J. D. Rioux, S. R. Brant, M. S. Silverberg, K. D. Taylor, M. M. Barmada, A. Bitton, T. Dassopoulos, L. W. Datta, T. Green, A. M. Griffiths, E. O. Kistner, M. T. Murtha, M. D. Regueiro, J. I. Rotter, L. P. Schumm, A. H. Steinhart, S. R. Targan, R. J. Xavier; NIDDK IBD Genetics Consortium, C. Libioulle, C. Sandor, M. Lathrop, J. Belaiche, O. Dewit, I. Gut, S. Heath, D. Laukens, M. Mni, P. Rutgeerts, A. Van Gossum, D. Zelenika, D. Franchimont, J.-P. Hugot, M. de Vos, S. Vermeire, E. Louis; Belgian-French IBD Consortium; Wellcome Trust Case Control Consortium, L. R. Cardon, C. A. Anderson, H. Drummond, E. Nimmo, T. Ahmad, N. J. Prescott, C. M. Onnie, S. A. Fisher, J. Marchini, J. Ghori, S. Bumpstead, R. Gwilliam, M. Tremelling, P. Deloukas, J. Mansfield, D. Jewell, J. Satsangi, C. G. Mathew, M. Parkes, M. Georges, M. J. Daly, Genome-wide association defines more than 30 distinct susceptibility loci for Crohn’s disease. Nat. Genet. 40, 955–962 (2008). 18587394 10.1038/NG.175 PMC2574810

[25] L. Jostins, S. Ripke, R. K. Weersma, R. H. Duerr, D. P. McGovern, K. Y. Hui, J. C. Lee, L. P. Schumm, Y. Sharma, C. A. Anderson, J. Essers, M. Mitrovic, K. Ning, I. Cleynen, E. Theatre, S. L. Spain, S. Raychaudhuri, P. Goyette, Z. Wei, C. Abraham, J.-P. Achkar, T. Ahmad, L. Amininejad, A. N. Ananthakrishnan, V. Andersen, J. M. Andrews, L. Baidoo, T. Balschun, P. A. Bampton, A. Bitton, G. Boucher, S. Brand, C. Büning, A. Cohain, S. Cichon, M. D’Amato, D. De Jong, K. L. Devaney, M. Dubinsky, C. Edwards, D. Ellinghaus, L. R. Ferguson, D. Franchimont, K. Fransen, R. Gearry, M. Georges, C. Gieger, J. Glas, T. Haritunians, A. Hart, C. Hawkey, M. Hedl, X. Hu, T. H. Karlsen, L. Kupcinskas, S. Kugathasan, A. Latiano, D. Laukens, I. C. Lawrance, C. W. Lees, E. Louis, G. Mahy, J. Mansfield, A. R. Morgan, C. Mowat, W. Newman, O. Palmieri, C. Y. Ponsioen, U. Potocnik, N. J. Prescott, M. Regueiro, J. I. Rotter, R. K. Russell, J. D. Sanderson, M. Sans, J. Satsangi, S. Schreiber, L. A. Simms, J. Sventoraityte, S. R. Targan, K. D. Taylor, M. Tremelling, H. W. Verspaget, M. De Vos, C. Wijmenga, D. C. Wilson, J. Winkelmann, R. J. Xavier, S. Zeissig, B. Zhang, C. K. Zhang, H. Zhao; International IBD Genetics Consortium (IIBDGC), M. S. Silverberg, V. Annese, H. Hakonarson, S. R. Brant, G. Radford-Smith, C. G. Mathew, J. D. Rioux, E. E. Schadt, M. J. Daly, A. Franke, M. Parkes, S. Vermeire, J. C. Barrett, J. H. Cho, Host–microbe interactions have shaped the genetic architecture of inflammatory bowel disease. Nature 491, 119–124 (2012). 23128233 10.1038/nature11582 PMC3491803

[26] C. Yao, D. Sakata, Y. Esaki, Y. Li, T. Matsuoka, K. Kuroiwa, Y. Sugimoto, S. Narumiya, Prostaglandin E2-EP4 signaling promotes immune inflammation through Th1 cell differentiation and Th17 cell expansion. Nat. Med. 15, 633–640 (2009). 19465928 10.1038/nm.1968

[27] C. Yao, T. Hirata, K. Soontrapa, X. Ma, H. Takemori, S. Narumiya, Prostaglandin E_2_ promotes Th1 differentiation via synergistic amplification of IL-12 signalling by cAMP and PI3-kinase. Nat. Commun. 4, 1685 (2013). 23575689 10.1038/ncomms2684 PMC3644078

[28] J. Lee, T. Aoki, D. Thumkeo, R. Siriwach, C. Yao, S. Narumiya, T cell–intrinsic prostaglandin E2-EP2/EP4 signaling is critical in pathogenic TH17 cell–driven inflammation. J. Allergy Clin. Immunol. 143, 631–643 (2019). 29935220 10.1016/j.jaci.2018.05.036 PMC6354914

[29] D. M. Kofler, A. Marson, M. Dominguez-Villar, S. Xiao, V. K. Kuchroo, D. A. Hafler, Decreased RORC-dependent silencing of prostaglandin receptor EP2 induces autoimmune Th17 cells. J. Clin. Invest. 124, 2513–2522 (2014). 24812667 10.1172/JCI72973 PMC4089462

[30] A. Tang, N. Li, X. Li, H. Yang, W. Wang, L. Zhang, G. Li, W. Xiong, J. Ma, S. Shen, Dynamic activation of the key pathways: Linking colitis to colorectal cancer in a mouse model. Carcinogenesis 33, 1375–1383 (2012). 22610167 10.1093/carcin/bgs183

[31] B.-H. Yang, S. Hagemann, P. Mamareli, U. Lauer, U. Hoffmann, M. Beckstette, L. Föhse, I. Prinz, J. Pezoldt, S. Suerbaum, T. Sparwasser, A. Hamann, S. Floess, J. Huehn, M. Lochner, Foxp3(+) T cells expressing RORγt represent a stable regulatory T-cell effector lineage with enhanced suppressive capacity during intestinal inflammation. Mucosal Immunol. 9, 444–457 (2016). 26307665 10.1038/mi.2015.74

[32] A. Schneider, Y. Guan, Y. Zhang, M. A. Magnuson, C. Pettepher, C. D. Loftin, R. Langenbach, R. M. Breyer, M. D. Breyer, Generation of a conditional allele of the mouse prostaglandin EP4 receptor. Genesis 40, 7–14 (2004). 15354288 10.1002/gene.20048

[33] M. A. M. Rogers, D. M. Aronoff, The influence of non-steroidal anti-inflammatory drugs on the gut microbiome. Clin. Microbiol. Infect. 22, 178.e1–178.e9 (2016). 10.1016/j.cmi.2015.10.003 PMC4754147 26482265

[34] X. Liang, K. Bittinger, X. Li, D. R. Abernethy, F. D. Bushman, G. A. FitzGerald, Bidirectional interactions between indomethacin and the murine intestinal microbiota. eLife 4, e08973 (2015). 26701907 10.7554/eLife.08973 PMC4755745

[35] Y. Yun, H.-N. Kim, S. E. Kim, Y. Chang, S. Ryu, H. Shin, S.-Y. Woo, H.-L. Kim, The effect of probiotics, antibiotics, and antipyretic analgesics on gut microbiota modification. J. Bacteriol. Virol. 47, 64 (2017).

[36] A. Beller, A. Kruglov, P. Durek, V. von Goetze, K. Werner, G. A. Heinz, J. Ninnemann, K. Lehmann, R. Maier, U. Hoffmann, R. Riedel, K. Heiking, J. Zimmermann, B. Siegmund, M.-F. Mashreghi, A. Radbruch, H.-D. Chang, Specific microbiota enhances intestinal IgA levels by inducing TGF-β in T follicular helper cells of Peyer’s patches in mice. Eur. J. Immunol. 50, 783–794 (2020). 32065660 10.1002/eji.201948474

[37] A. Couturier-Maillard, T. Secher, A. Rehman, S. Normand, A. De Arcangelis, R. Haesler, L. Huot, T. Grandjean, A. Bressenot, A. Delanoye-Crespin, O. Gaillot, S. Schreiber, Y. Lemoine, B. Ryffel, D. Hot, G. Nùñez, G. Chen, P. Rosenstiel, M. Chamaillard, NOD2-mediated dysbiosis predisposes mice to transmissible colitis and colorectal cancer. J. Clin. Invest. 123, 700–711 (2013). 23281400 10.1172/JCI62236 PMC3561825

[38] V. De Preter, K. Machiels, M. Joossens, I. Arijs, C. Matthys, S. Vermeire, P. Rutgeerts, K. Verbeke, Faecal metabolite profiling identifies medium-chain fatty acids as discriminating compounds in IBD. Gut 64, 447–458 (2015). 24811995 10.1136/gutjnl-2013-306423

[39] I. Ahmed, R. Greenwood, B. Costello, N. Ratcliffe, C. S. Probert, Investigation of faecal volatile organic metabolites as novel diagnostic biomarkers in inflammatory bowel disease. Aliment. Pharmacol. Ther. 43, 596–611 (2016). 26806034 10.1111/apt.13522

[40] A. B. Granado-Serrano, M. Martín-Garí, V. Sánchez, M. Riart Solans, R. Berdún, I. A. Ludwig, L. Rubió, E. Vilaprinyó, M. Portero-Otín, J. C. E. Serrano, Faecal bacterial and short-chain fatty acids signature in hypercholesterolemia. Sci. Rep. 9, 1772 (2019). 30742005 10.1038/s41598-019-38874-3 PMC6370822

[41] X. Zhu, Y. Zhou, Y. Wang, T. Wu, X. Li, D. Li, Y. Tao, Production of high-concentration n-caproic acid from lactate through fermentation using a newly isolated *Ruminococcaceae* bacterium CPB6. Biotechnol. Biofuels 10, 102 (2017). 28439295 10.1186/s13068-017-0788-y PMC5399333

[42] C.-J. Guo, B. M. Allen, K. J. Hiam, D. Dodd, W. V. Treuren, S. Higginbottom, K. Nagashima, C. R. Fischer, J. L. Sonnenburg, M. H. Spitzer, M. A. Fischbach, Depletion of microbiome-derived molecules in the host using *Clostridium* genetics. Science 366, eaav1282 (2019). 31831639 10.1126/science.aav1282 PMC7141153

[43] C.-M. Sun, J. A. Hall, R. B. Blank, N. Bouladoux, M. Oukka, J. R. Mora, Y. Belkaid, Small intestine lamina propria dendritic cells promote de novo generation of Foxp3 T reg cells via retinoic acid. J. Exp. Med. 204, 1775–1785 (2007). 17620362 10.1084/jem.20070602 PMC2118682

[44] M. Kim, C. Galan, A. A. Hill, W.-J. Wu, H. Fehlner-Peach, H. W. Song, D. Schady, M. L. Bettini, K. W. Simpson, R. S. Longman, D. R. Littman, G. E. Diehl, Critical role for the microbiota in CX_3_CR1^+^ intestinal mononuclear phagocyte regulation of intestinal T cell responses. Immunity 49, 151–163.e5 (2018). 29980437 10.1016/j.immuni.2018.05.009 PMC6051886

[45] J. L. Coombes, K. R. R. Siddiqui, C. V. Arancibia-Cárcamo, J. Hall, C.-M. Sun, Y. Belkaid, F. Powrie, A functionally specialized population of mucosal CD103^+^ DCs induces Foxp3^+^ regulatory T cells via a TGF-β and retinoic acid-dependent mechanism. J. Exp. Med. 204, 1757–1764 (2007). 17620361 10.1084/jem.20070590 PMC2118683

[46] C. Nakahashi-Oda, K. G. S. Udayanga, Y. Nakamura, Y. Nakazawa, N. Totsuka, H. Miki, S. Iino, S. Tahara-Hanaoka, S. Honda, K. Shibuya, A. Shibuya, Apoptotic epithelial cells control the abundance of T_reg_ cells at barrier surfaces. Nat. Immunol. 17, 441–450 (2016). 26855029 10.1038/ni.3345

[47] Y. Tanaka, H. Nagashima, K. Bando, L. Lu, A. Ozaki, Y. Morita, S. Fukumoto, N. Ishii, S. Sugawara, Oral CD103^−^CD11b^+^ classical dendritic cells present sublingual antigen and induce Foxp3^+^ regulatory T cells in draining lymph nodes. Mucosal Immunol. 10, 79–90 (2017). 27166558 10.1038/mi.2016.46

[48] A. Metidji, S. A. Rieder, D. D. Glass, I. Cremer, G. A. Punkosdy, E. M. Shevach, IFN-α/β receptor signaling promotes regulatory T cell development and function under stress conditions. J. Immunol. 194, 4265–4276 (2015). 25795758 10.4049/jimmunol.1500036 PMC4402260

[49] Y. Matsumoto, Y. Nakanishi, T. Yoshioka, Y. Yamaga, T. Masuda, Y. Fukunaga, M. Sono, T. Yoshikawa, M. Nagao, O. Araki, S. Ogawa, N. Goto, Y. Hiramatsu, R. M. Breyer, A. Fukuda, H. Seno, Epithelial EP4 plays an essential role in maintaining homeostasis in colon. Sci. Rep. 9, 15244 (2019). 31645712 10.1038/s41598-019-51639-2 PMC6811535

[50] M. Xu, M. Pokrovskii, Y. Ding, R. Yi, C. Au, O. J. Harrison, C. Galan, Y. Belkaid, R. Bonneau, D. R. Littman, c-MAF-dependent regulatory T cells mediate immunological tolerance to a gut pathobiont. Nature 554, 373–377 (2018). 29414937 10.1038/nature25500 PMC5814346

[51] L. Maier, M. Pruteanu, M. Kuhn, G. Zeller, A. Telzerow, E. E. Anderson, A. R. Brochado, K. C. Fernandez, H. Dose, H. Mori, K. R. Patil, P. Bork, A. Typas, Extensive impact of non-antibiotic drugs on human gut bacteria. Nature 555, 623–628 (2018). 29555994 10.1038/nature25979 PMC6108420

[52] K. N. Khan, M. Kitajima, N. Yamaguchi, A. Fujishita, M. Nakashima, T. Ishimaru, H. Masuzaki, Role of prostaglandin E2 in bacterial growth in women with endometriosis. Hum. Reprod. 27, 3417–3424 (2012). 23001777 10.1093/humrep/des331

[53] C. Panea, A. M. Farkas, Y. Goto, S. Abdollahi-Roodsaz, C. Lee, B. Koscsó, K. Gowda, T. M. Hohl, M. Bogunovic, I. I. Ivanov, Intestinal monocyte-derived macrophages control commensal-specific Th17 responses. Cell Rep. 12, 1314–1324 (2015). 26279572 10.1016/j.celrep.2015.07.040 PMC4567384

[54] A. Gangaplara, C. Martens, E. Dahlstrom, A. Metidji, A. S. Gokhale, D. D. Glass, M. Lopez-Ocasio, R. Baur, K. Kanakabandi, S. F. Porcella, E. M. Shevach, Type I interferon signaling attenuates regulatory T cell function in viral infection and in the tumor microenvironment. PLOS Pathog. 14, e1006985 (2018). 29672594 10.1371/journal.ppat.1006985 PMC5929570

[55] Y. Li, C. Soendergaard, F. H. Bergenheim, D. M. Aronoff, G. Milne, L. B. Riis, J. B. Seidelin, K. B. Jensen, O. H. Nielsen, COX-2-PGE2 signaling impairs intestinal epithelial regeneration and associates with TNF inhibitor responsiveness in ulcerative colitis. EBioMedicine 36, 497–507 (2018). 30190207 10.1016/j.ebiom.2018.08.040 PMC6197735

[56] D. Maseda, E. M. Johnson, L. E. Nyhoff, B. Baron, F. Kojima, A. J. Wilhelm, M. R. Ward, J. G. Woodward, D. D. Brand, L. J. Crofford, mPGES1-dependent prostaglandin E2(PGE2) controls antigen-specific Th17 and Th1 responses by regulating T autocrine and paracrine PGE2 production. J. Immunol. 200, 725–736 (2018). 29237778 10.4049/jimmunol.1601808 PMC5760456

[57] K. Gerasimidis, K. Bryden, X. Chen, E. Papachristou, A. Verney, M. Roig, R. Hansen, B. Nichols, R. Papadopoulou, A. Parrett, The impact of food additives, artificial sweeteners and domestic hygiene products on the human gut microbiome and its fibre fermentation capacity. Eur. J. Nutr. 59, 3213–3230 (2020). 31853641 10.1007/s00394-019-02161-8 PMC7501109

[58] R. B. Sarma-Rupavtarm, Z. Ge, D. B. Schauer, J. G. Fox, M. F. Polz, Spatial distribution and stability of the eight microbial species of the altered schaedler flora in the mouse gastrointestinal tract. Appl. Environ. Microbiol. 70, 2791–2800 (2004). 15128534 10.1128/AEM.70.5.2791-2800.2004 PMC404395

[59] C. Tang, S. Kakuta, K. Shimizu, M. Kadoki, T. Kamiya, T. Shimazu, S. Kubo, S. Saijo, H. Ishigame, S. Nakae, Y. Iwakura, Suppression of IL-17F, but not of IL-17A, provides protection against colitis by inducing T_reg_ cells through modification of the intestinal microbiota. Nat. Immunol. 19, 755–765 (2018). 29915298 10.1038/s41590-018-0134-y

[60] K. Yoshikawa, C. Kurihara, H. Furuhashi, T. Takajo, K. Maruta, Y. Yasutake, H. Sato, K. Narimatsu, Y. Okada, M. Higashiyama, C. Watanabe, S. Komoto, K. Tomita, S. Nagao, S. Miura, H. Tajiri, R. Hokari, Psychological stress exacerbates NSAID-induced small bowel injury by inducing changes in intestinal microbiota and permeability via glucocorticoid receptor signaling. J. Gastroenterol. 52, 61–71 (2017). 27075753 10.1007/s00535-016-1205-1

[61] N. Larsen, F. K. Vogensen, F. W. J. van den Berg, D. S. Nielsen, A. S. Andreasen, B. K. Pedersen, W. A. Al-Soud, S. J. Sørensen, L. H. Hansen, M. Jakobsen, Gut microbiota in human adults with type 2 diabetes differs from non-diabetic adults. PLOS ONE 5, e9085 (2010). 20140211 10.1371/journal.pone.0009085 PMC2816710

[62] M. Barman, D. Unold, K. Shifley, E. Amir, K. Hung, N. Bos, N. Salzman, Enteric salmonellosis disrupts the microbial ecology of the murine gastrointestinal tract. Infect. Immun. 76, 907–915 (2008). 18160481 10.1128/IAI.01432-07 PMC2258829

[63] M. Vital, C. R. Penton, Q. Wang, V. B. Young, D. A. Antonopoulos, M. L. Sogin, H. G. Morrison, L. Raffals, E. B. Chang, G. B. Huffnagle, T. M. Schmidt, J. R. Cole, J. M. Tiedje, A gene-targeted approach to investigate the intestinal butyrate-producing bacterial community. Microbiome 1, 8 (2013). 24451334 10.1186/2049-2618-1-8 PMC4126176

[64] J. Pollock, D. L. Gally, L. Glendinning, R. Tiwari, M. R. Hutchings, J. G. M. Houdijk, Analysis of temporal fecal microbiota dynamics in weaner pigs with and without exposure to enterotoxigenic *Escherichia coli*. J. Anim. Sci. 96, 3777–3790 (2018). 29982429 10.1093/jas/sky260 PMC6127793

[65] M. Martin, Cutadapt removes adapter sequences from high-throughput sequencing reads. EMBnet.J. 17, 10–12 (2011).

[66] B. J. Callahan, P. J. McMurdie, M. J. Rosen, A. W. Han, A. J. A. Johnson, S. P. Holmes, DADA2: High-resolution sample inference from Illumina amplicon data. Nat. Methods 13, 581–583 (2016). 27214047 10.1038/nmeth.3869 PMC4927377

[67] E. Bolyen, J. R. Rideout, M. R. Dillon, N. A. Bokulich, C. C. Abnet, G. A. Al-Ghalith, H. Alexander, E. J. Alm, M. Arumugam, F. Asnicar, Y. Bai, J. E. Bisanz, K. Bittinger, A. Brejnrod, C. J. Brislawn, C. T. Brown, B. J. Callahan, A. M. Caraballo-Rodríguez, J. Chase, E. K. Cope, R. Da Silva, C. Diener, P. C. Dorrestein, G. M. Douglas, D. M. Durall, C. Duvallet, C. F. Edwardson, M. Ernst, M. Estaki, J. Fouquier, J. M. Gauglitz, S. M. Gibbons, D. L. Gibson, A. Gonzalez, K. Gorlick, J. Guo, B. Hillmann, S. Holmes, H. Holste, C. Huttenhower, G. A. Huttley, S. Janssen, A. K. Jarmusch, L. Jiang, B. D. Kaehler, K. B. Kang, C. R. Keefe, P. Keim, S. T. Kelley, D. Knights, I. Koester, T. Kosciolek, J. Kreps, M. G. I. Langille, J. Lee, R. Ley, Y.-X. Liu, E. Loftfield, C. Lozupone, M. Maher, C. Marotz, B. D. Martin, D. McDonald, L. J. McIver, A. V. Melnik, J. L. Metcalf, S. C. Morgan, J. T. Morton, A. T. Naimey, J. A. Navas-Molina, L. F. Nothias, S. B. Orchanian, T. Pearson, S. L. Peoples, D. Petras, M. L. Preuss, E. Pruesse, L. B. Rasmussen, A. Rivers, M. S. Robeson, P. Rosenthal, N. Segata, M. Shaffer, A. Shiffer, R. Sinha, S. J. Song, J. R. Spear, A. D. Swafford, L. R. Thompson, P. J. Torres, P. Trinh, A. Tripathi, P. J. Turnbaugh, S. Ul-Hasan, J. J. J. van der Hooft, F. Vargas, Y. Vázquez-Baeza, E. Vogtmann, M. von Hippel, W. Walters, Y. Wan, M. Wang, J. Warren, K. C. Weber, C. H. D. Williamson, A. D. Willis, Z. Z. Xu, J. R. Zaneveld, Y. Zhang, Q. Zhu, R. Knight, J. G. Caporaso, Reproducible, interactive, scalable and extensible microbiome data science using QIIME 2. Nat. Biotechnol. 37, 852–857 (2019). 31341288 10.1038/s41587-019-0209-9 PMC7015180

[68] F. Pedregosa, G. Varoquaux, A. Gramfort, V. Michel, B. Thirion, O. Grisel, M. Blondel, P. Prettenhofer, R. Weiss, V. Dubourg, J. Vanderplas, A. Passos, D. Cournapeau, M. Brucher, M. Perrot, É. Duchesnay, Scikit-learn: Machine learning in Python. J. Mach. Learn. Res. 12, 2825–2830 (2011).

[69] K. Katoh, D. M. Standley, MAFFT multiple sequence alignment software version 7: Improvements in performance and usability. Mol. Biol. Evol. 30, 772–780 (2013). 23329690 10.1093/molbev/mst010 PMC3603318

[70] P. J. McMurdie, S. Holmes, phyloseq: An R package for reproducible interactive analysis and graphics of microbiome census data. PLOS ONE 8, e61217 (2013). 23630581 10.1371/journal.pone.0061217 PMC3632530

[71] C. Lozupone, R. Knight, UniFrac: A new phylogenetic method for comparing microbial communities. Appl. Environ. Microbiol. 71, 8228–8235 (2005). 16332807 10.1128/AEM.71.12.8228-8235.2005 PMC1317376

